# A modelling study of right ventricular growth with valvular regurgitation

**DOI:** 10.1007/s10237-026-02113-2

**Published:** 2026-08-05

**Authors:** Debao Guan, Hao Gao

**Affiliations:** 1 School of Control Science and Engineering, Shandong University, Jinan, China; 2 School of Mathematics and Statistics, University of Glasgow, Glasgow, UK

**Keywords:** Computational modelling, Right ventricle, Valvular regurgitation, Kinematic growth, Eccentric growth

## Abstract

Right ventricular (RV) dysfunction due to pulmonary and tricuspid valve regurgitation remains understudied despite its critical role in adverse cardiac outcomes. We present a biventricular computational model that integrates regurgitant valves in the RV with a kinematic growth framework. Updated reference configurations are used to allow saturated growth in each growth cycle. Acute regurgitation scenarios and long-term adaptation are modelled to quantify structural and functional adaptations in the RV and their further impacts on left ventricular (LV) performance. Results demonstrate that persistent regurgitation drives dominant eccentric growth in the RV, leading to severe cavity dilation, septal displacement, and impaired LV filling and systolic function. Simulations incorporating both eccentric and concentric growth reveal a limited compensatory role for concentric thickening, even under severe volume overload. The simulated haemodynamic and functional responses are broadly consistent with clinical observations and capture clinically plausible trajectories of RV growth under sustained regurgitation. These findings suggest that biomechanical modelling of myocardial adaptation could provide mechanistic insights into RV adaptation under severe valve regurgitation, and may support clinical decision-making regarding RV failure once fully validated. Future work should focus on validating myocardial growth laws using experimental and clinical data, and extending the framework to patient-specific scenarios for predictive modelling of RV dysfunction due to valve regurgitation.

## Introduction

Computational modelling of cardiac disease (Sel et al. 2024; Trayanova and Prakosa 2024), either mechanics-driven or data-driven, calibrated with individual patients’ data, can provide clinically relevant quantification which is otherwise not available, i.e. myofibre stress, and to predict the short-term and long-term evolutions of those quantities, such as heart structural and functional adaptations, as well as the responses to interventions. Various cardiac models have been developed over the last several decades (Chabiniok et al. 2016; Mangion et al. 2018; Rodero et al. 2023), ranging from lumped-parameter models to idealised and generic models, to population-average and patient-specific models built upon existing clinical data, from single ventricle to bi-ventricle, 4-chamber whole heart with blood-myocardium-valve interaction (Feng et al. 2024; Davey et al. 2024), further coupled with electrophysiology (Zingaro et al. 2024).

Current state-of-the-art cardiac biomechanics models mostly focus on heart dynamics and its vasculature to predict current states or immediate outcomes to external actions; therefore, their predictive capability of disease progression or patient recovery is limited due to the “snapshot” nature. In addition, modelling right ventricle (RV) dynamics is less extensive than the LV despite its equal importance as the left heart (Morgan et al. 2020), partly because of the RV’s complex anatomy and the strong mechanical coupling between the two ventricles. An open question is how right-sided valve regurgitation, alone or in combination, can drive progressive RV growth and remodelling.

Clinically, chronic pulmonary regurgitation (PR), often seen after surgical valvotomy or repaired tetralogy of Fallot (rTOF), and tricuspid regurgitation (TR) affecting about 0.5% of the adult population (Topilsky et al. 2019), can both drive adverse RV growth and remodelling (G&R), cause RV progressive dilation, and eventually lead to RV pump function failure if persistent. Quantification of tricuspid valve (TV) function and RV’s long-term remodelling requires a comprehensive biomechanics study of TV dynamics, as has been done in the aortic valve and the mitral valve. The first three-dimensional TV model was developed in 2010 (Stevanella et al. 2010) based on experimental data from porcine and human valves, which highlighted the importance of the collagen network. Since then, modelling studies on TV have been increasingly reported. For example, Dabiri et al. (2019) investigated the effects of mitral clip position on treating TR using fluid–structure interaction analysis; Laurence et al. (2020) studied how different pathological conditions affected TV dynamics such as papillary muscle movement, annulus ring dilation and chordae rupture; Mathur et al. (2022) simulated surgical annuloplasty and transcatheter edge-to-edge repair in a high-fidelity TV model from a beating human heart; Very recently, Moola et al. (2026) developed an automated patient-specific TV modelling pipeline to achieve direct image-to-analysis. Although modelling TV alone provides useful information on its dynamics, it is also paramount to understand its impact on RV function (Morgan et al. 2020). Very limited studies exist in modelling TV and RV together, except for some recent 4-chamber hearts (Feng et al. 2024; Davey et al. 2024; Fedele et al. 2023).

Chronic pathological PR also results in chronic RV volume overload with ventricular dilation and dysfunction (Fathallah and Krasuski 2017; Akazawa et al. 2021). Pulmonary valve (PV) replacement has yielded inconsistent outcomes in patient survival (Vliegen et al. 2002) partially due to the difficulty of accurately assessing RV function non-invasively. In addition, the surgery to replace PV carries a perioperative risk, and bioprosthetic valves are further subject to ongoing degeneration and endocarditis risk. Therefore, to improve patient selection and timing of PV replacement, it would be valuable to have a means of quantifying RV function in the presence of PR and the ability to predict the effect on RV mechanics following PV replacement. Gusseva et al. (2021a) modelled PV replacement in rTOF patients using a lumped-parameter model, and they found a linear relationship between the model-predicted RV contractility post PV replacement and RV outflow obstruction resistance (Gusseva et al. 2021b). Although PR may be well tolerated for prolonged periods, optimal timing and selection for TV intervention remain uncertain because of the higher risk of mortality than surgically repairing other cardiac valves. Those challenges motivate biomechanical modelling frameworks focusing on RV that can quantify RV function and its adaptation due to valve dysfunction, and ultimately help identify patients most likely to benefit from pulmonary and tricuspid valve corrections.

The increased volume load on the RV due to PR and TR usually leads to elevated myocardial stress and strain, which could potentially trigger a cascade of cellular and extracellular matrix remodelling processes (Lyon et al. 2015; Khassafi et al. 2023). As a result, cardiomyocytes may undergo hypertrophy, and activated fibroblasts could further contribute to changes in the extracellular matrix composition, leading to fibrosis. These changes aim to normalise myocardial stress and stretch, and maintain cardiac function, but could potentially result in adverse remodelling, impairing the heart’s pump efficiency over time (Maillet et al. 2013; Lyon et al. 2015). Other factors, such as neurohormonal stimulation, inflammatory cytokines, etc., together with mechanical factors, modulate various cellular responses, including changes in gene expression, protein synthesis, sarcomere assembly, and cell metabolism, all of which will contribute to the development and progression of cardiac hypertrophy (Maillet et al. 2013). Mathematical modelling of cardiac G&R within the nonlinear finite element framework has been developed for several decades, including the kinematic growth theory (Rodriguez et al. 1994) and the constrained mixture theory (Humphrey and Rajagopal 2002). Both approaches have been successfully applied to study how the heart will adapt its structure and function (Göktepe et al. 2010; Genet et al. 2016; Gebauer et al. 2023) driven by an imbalanced biomechanical environment, mainly into two categories: the eccentric growth (e.g. adding sarcomeres in series) and the concentric growth (e.g. adding sarcomeres in parallel). Recent studies have studied LV thinning and fibrosis following myocardial infarction (Guan et al. 2025), and LV G&R due to aortic stenosis and mitral regurgitation by further considering the baroreflex feedback loop (Mehri and Wenk 2025). On RV G&R, Avazmohammadi et al. (2019) studied the volumetric growth of a rodent RV due to pulmonary arterial hypertension (PAH) and the re-orientation of myofibres. A recent summary on modelling RV G&R due to PAH using computational approaches can be found in (Odeigah et al. 2022).

In clinical literature, remodelling often refers to both geometric and functional adaptation, as well as microstructural changes. In this study, we define *growth* as the change in myocardial mass or volume, and *remodelling* as microstructural alterations, such as cross-link formation and fibre reorientation. In this study, we present a biventricular model that focuses on RV biomechanics and its growth, as a proof-of-concept framework to assess and predict RV dysfunction induced by PV and TV regurgitation. This work extends our previous study (Guan and Gao 2025) by providing a detailed mathematical formulation of the growth framework. Specifically, we add: (1) RV eccentric growth across a range of regurgitation scenarios; (2) hybrid growth in the RV and the biventricular setting; and (3) an expanded analysis of RV–LV interdependence. This study aims to establish a mechanistic framework to investigate how different regurgitation conditions drive ventricular growth and functional progression, rather than to develop a patient-specific predictive model of RV growth, or propose a new growth formulation.

## Method

### The biventricular model with RV regurgitation

To develop an RV model suitable for simulating both PV and TV regurgitation, we first constructed a biventricular model with a right ventricular outflow tract (RVOT) in which the pulmonary valve sits. A prior in vivo magnetic resonance imaging (MRI) study of a healthy volunteer (33 years, female) with no prior history of cardiovascular disease was chosen. The MRI study was approved by the National Research Ethics Service, and written consent was obtained before the imaging study. Details of this MRI study can be found in Gao et al. (2017).

**Fig. 1:**
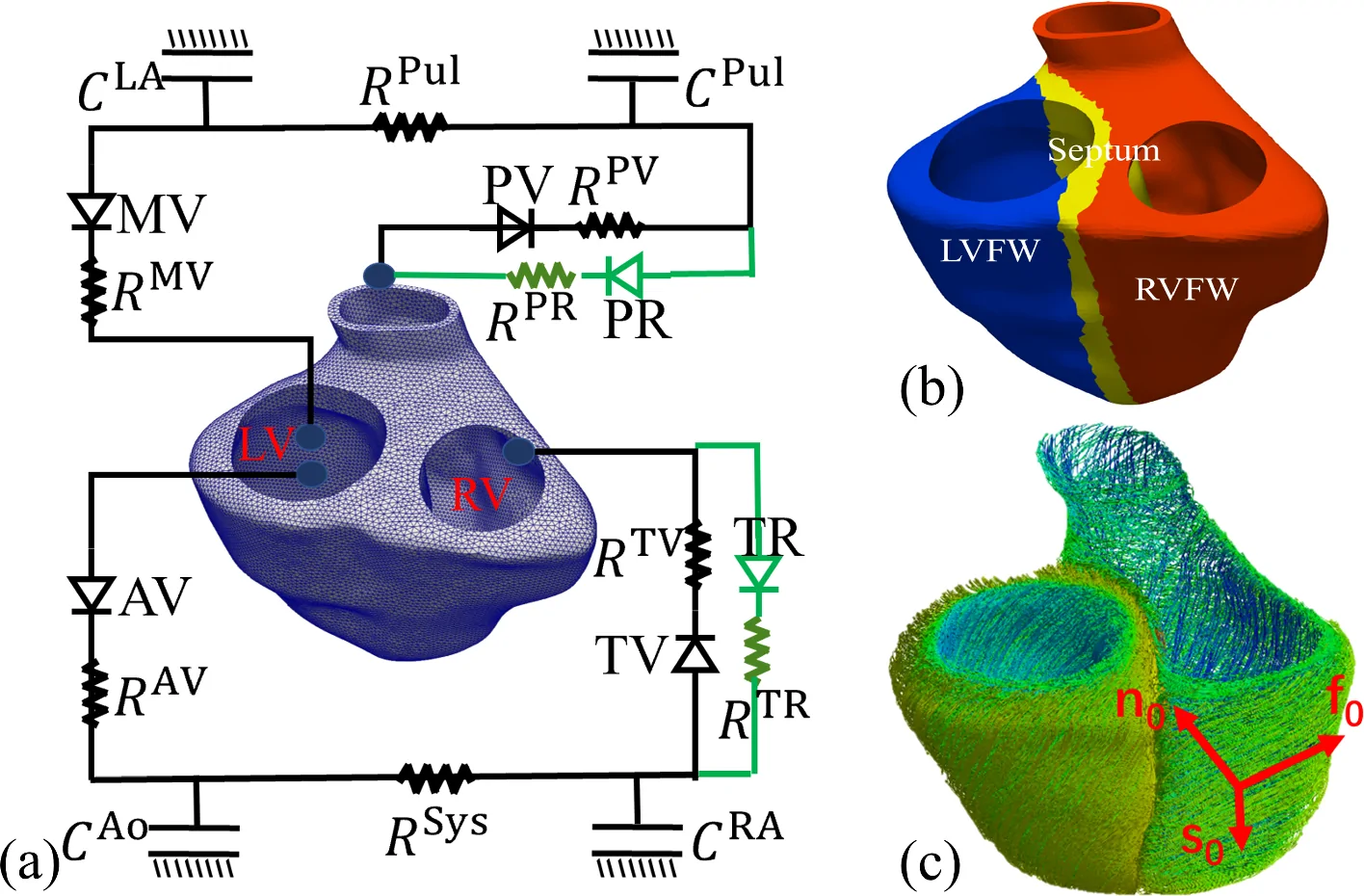
Schematic illustration of the human biventricle model coupled to systemic circulation and pulmonary circulation (**a**), in which *R* represents the resistance between cavities, *C* represents the compliance of a cavity, and the diode controls the unidirectional flow. MV: mitral valve, AV: aortic valve, RA: right atrium, TV: tricuspid valve, PV: pulmonary valve, LA: left atrium, Ao: aorta, Sys: systemic circulation, Pul: pulmonary circulation, PR: PV regurgitation, TR: TV regurgitation. The bi-ventricle model is further divided into three parts (**b**): the left ventricular free wall (LVFW) in blue, the right ventricular free wall (RVFW) in red, and the septum in yellow. **(c)** The rule-based myofibre structure where $\textbf{f}_0,\,\textbf{s}_0,\,\textbf{n}_0$ are the unit myofibre, sheet, and sheet-normal directions in the reference configuration

The biventricular geometry of this healthy volunteer was constructed using both short-axis and long-axis cine images. The unloaded state was chosen at the early-diastolic phase when the ventricular pressure is at its lowest in both the LV and RV cavities. Ventricular wall boundaries were manually delineated at each image plane, including LV endocardium, RV endocardium and the epicardium. The LV endocardial surface, the RV endocardial surface and the epicardial surface were reconstructed from those boundary point clouds for the geometry generation, as shown in Fig. 1a. The heart was further divided into three regions: the LV free wall (LVFW), the RV free wall (RVFW) and the septum, see Fig. 1b. Lumped parameter models of the systemic and pulmonary circulations were further incorporated to ensure physiologically accurate pressure boundary conditions for both the LV and RV, as shown in Fig. 1a. To simulate regurgitation in the RV, one-directional valves were introduced: a pulmonary regurgitation valve parallel to the PV, and a tricuspid regurgitation valve parallel to the TV. The resistance of each valve determines the regurgitation severity with a lower resistance corresponding to a more severe regurgitation. These two regurgitation valves allow for simulations of isolated PR and TR, or combined P&TR, and their long-term effects on RV function through growth and remodelling.

The Laplace-Dirichlet method introduced in the work of Doste et al. (2019) was adopted here to generate a rule-based myofibre architecture, see Fig. 1c, with the fibre rotation angles varying linearly from − 60$^\circ$ at epicardium to 60$^\circ$ at endocardium in the LV, and from 90$^\circ$ on the RV endocardium to $-25^{o}$ on the RV epicardium. For the RVOT, the angle on the inner surface is 90$^\circ$ and 0$^\circ$ on the outer surface.

### Material model of the myocardium

The passive behaviour of the myocardium is characterised by an invariant-based strain energy function from Guan et al. (2019) by assuming the myocardium is anisotropic, hyperelastic and nearly incompressible, following the material model developed in Holzapfel and Ogden (2009). It is given by

1 $$\begin{aligned} \begin{aligned} W^\text {p}=&\frac{a}{2b}\{\exp [b(I_1-3)]-1\} \\&+\frac{a_\text {f}}{2b_\text {f}} \{\exp [b_\text {f}( I^*_\text {4f}-1)^2]-1\} \\& +\frac{a_\text {n}}{2b_\text {n}} \{\exp [b_\text {n}( I^*_\text {4n}-1)^2]-1\} \\& +\frac{a_\text {fs}}{2b_\text {fs}}\{\exp (b_{\text {fs}}I_{8\text {fs}}^2)-1\}, \end{aligned} \end{aligned}$$

where *a*, $a_{\text {f}}$, $a_\text {n}$, $a_\text {fs}$, *b*, $b_{\text {f}}$, $b_\text {n}$, and $b_\text {fs}$ are material parameters, and

$$\begin{aligned}&I_1 = \text {trace}(\textbf{F}^\text {T} \textbf{F}),\,\, I_\text {4f}=\textbf{F}\textbf{f}_0 \cdot \textbf{F}\textbf{f}_0,\,\, I_\text {4n}=\textbf{F}\boldsymbol{\textrm{n}}_0\cdot \textbf{F}\boldsymbol{\textrm{n}}_0,\,\\&\quad I_\text {8fs}=\textbf{F}\textbf{f}_0\cdot \textbf{F}\boldsymbol{\textrm{s}}_0 \end{aligned}$$

in which $\textbf{f}_0,\,\textbf{s}_0,\,\textbf{n}_0$ are the unit myofibre, sheet, and sheet-normal directions in the reference configuration, with $\textbf{F}$ the deformation gradient. The modified invariants in (1) are defined as

$$\begin{aligned} I^*_\text {4f}=\text {max}(I_\text {4f},1) \,\,\text {and}\,\, I^*_\text {4n}=\text {max}(I_\text {4n},1) \end{aligned}$$

so that only stretched fibres can contribute to the passive response. The total passive stress is defined as

2 $$\begin{aligned} \boldsymbol{\sigma }^{{\mathrm{p}}} = {\mathbf{F}}\frac{{\partial W}}{{\partial {\mathbf{F}}}} - p{\mathbf{I}} = & \,2a\exp [b(I_{1} - 3)]{\mathbf{B}} - p{\mathbf{I}} \\ & + 2a_{{\mathrm{f}}} (I_{{{\mathrm{4f}}}}^{*} - 1)\exp [b_{{\mathrm{f}}} (I_{{{\mathrm{4f}}}}^{*} - 1)^{2} ]{\mathbf{f}} \otimes {\mathbf{f}} \\ & + 2a_{{\mathrm{n}}} (I_{{{\mathrm{4n}}}}^{*} - 1)\exp [b_{{\mathrm{n}}} (I_{{{\mathrm{4n}}}}^{*} - 1)^{2} ]{\mathbf{n}} \otimes {\mathbf{n}} \\ & + a_{{{\mathrm{fs}}}} I_{{8{\mathrm{fs}}}} \exp (b_{{{\mathrm{fs}}}} I_{{8{\mathrm{fs}}}}^{2} )({\mathbf{f}} \otimes {\mathbf{s}} + {\mathbf{n}} \otimes {\mathbf{f}}), \\ \end{aligned}$$

in which $\textbf{B}=\textbf{F}\textbf{F}^\text {T}$, $\textbf{f}=\textbf{F}\textbf{f}_0$, $\boldsymbol{\textrm{s}}=\textbf{F}\boldsymbol{\textrm{s}}_0$, $\boldsymbol{\textrm{n}}=\textbf{F}\boldsymbol{\textrm{n}}_0$, *p* is the Lagrange multiplier to enforce incompressibility, and $\boldsymbol{\textrm{I}}$ is the identity matrix.

Following the additive approach, the total Cauchy stress of the myocardium is given by

3 $$\begin{aligned} \boldsymbol{{\sigma }} = \boldsymbol{{\sigma }}^\text {p} + \boldsymbol{{\sigma }}^\text {a}, \end{aligned}$$

where $\boldsymbol{{\sigma }}^\text {a}$ is the active stress tensor, which is characterized by a hybrid active contraction model proposed in Guan et al. (2022a),

4 $$\begin{aligned} \boldsymbol{{\sigma }}^\text {a} = K^p_a (1-\Lambda ^a) \, \frac{\eta \lambda _\text {f} - 1}{I_\text {4f}}{\textbf{f}} \otimes {\textbf{f}}, \end{aligned}$$

where $\lambda _\text {f} = \sqrt{I_\text {4f}}$, $K^p_a$ and η are constants, and $\Lambda ^a$ is a function of normalized intracellular calcium transient ($\text {Ca}$), such that

5 $$\begin{aligned} \Lambda ^a =\frac{1}{1+\mathcal {F}( \text {Ca})(\xi -1)}, \end{aligned}$$

in which $\xi =1/\Lambda ^\text {min}$, and the intracellular calcium transient is prescribed with the profile from Guan et al. (2022a). The value of $\Lambda ^\text {min}$ represents the minimum stretch ratio that a functional myocyte can achieve, defined as

6 $$\begin{aligned}\Lambda ^\text {min}(\lambda _\text {f},\dot{\lambda _\text {f}}) \nonumber = {\left\{ \begin{array}{ll} (\kappa _1 \lambda _\text {f}^2+\kappa _2 \lambda _\text {f}+\kappa _3)\frac{1+\kappa _4}{1+\kappa _4\,e^{\kappa _5 \dot{\lambda _\text {f}}}} \quad & \text {for} \quad \vartheta _1\vartheta _2 \le 1, \\ 1, & \text {otherwise}, \end{array}\right. } \end{aligned}$$

where $\kappa _1,..., \kappa _5$ are positive parameters, and $\dot{\lambda _\text {f}} = d \lambda _\text {f} / {d t}$. The function $\mathcal {F}(\cdot )$ in (5) controls the contraction behaviour,

7 $$\begin{aligned} \mathcal {F}({\text {Ca}})=1+\frac{2}{\pi }\arctan (\beta \ln { \text {[Ca]}}), \end{aligned}$$

where β is a constant. Details of this hybrid contraction model can be found in Guan et al. (2022a).

### Myocardial growth with update reference configuration

**Fig. 2:**
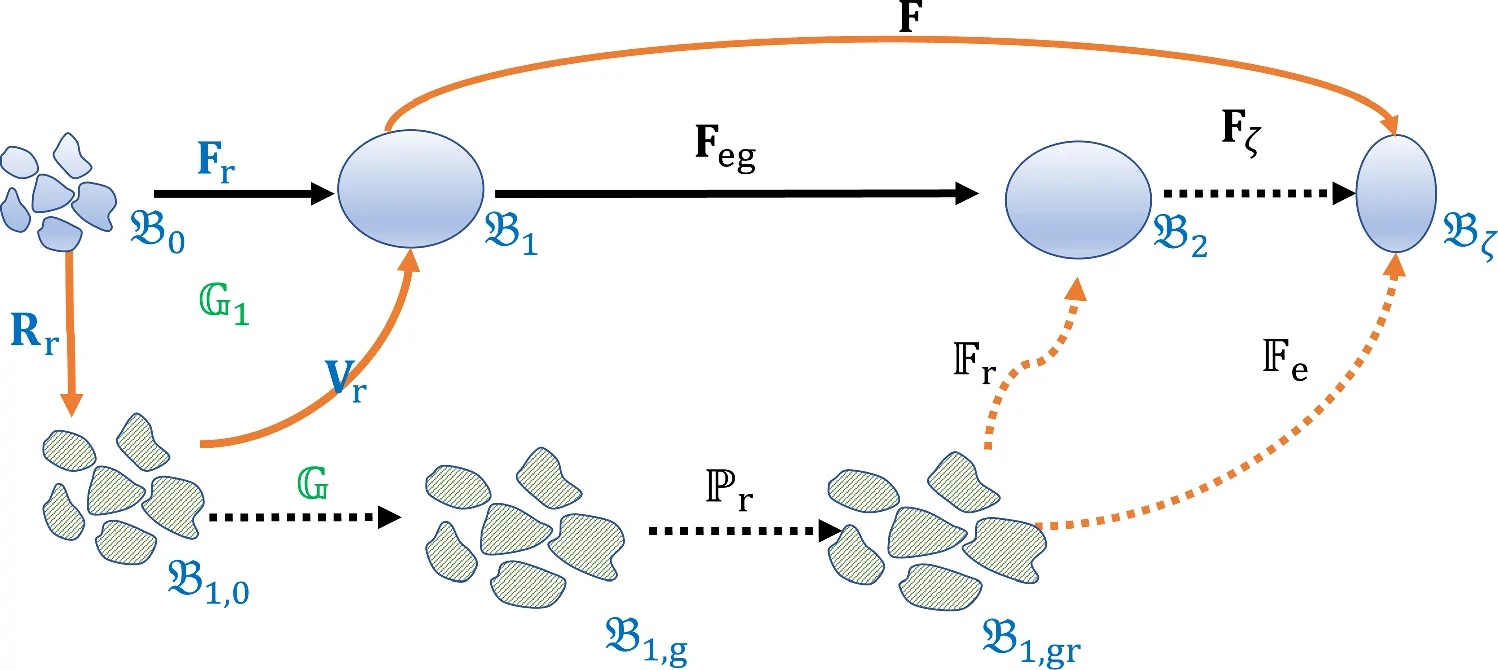
Schematic illustration of myocardial growth and remodelling in the updated reference configuration with non-zero residual stress. The current configuration of the biventricular model, denoted as $\mathfrak {B}_1$, can be reverted to a fictitious incompatible state, labelled as ${\mathfrak {B}}_{0}$ through the application of the residual elastic deformation tensor $\textbf{F}_\text {r}$. By applying $\boldsymbol{\textrm{R}}_\text {r}$ to ${\mathfrak {B}}_{0}$, it leads to another zero-stress incompatible configuration $\mathfrak {B}_{1,0}$. The left stretch tensor $\textbf{V}_\text {r}$ provides a mapping from $\mathfrak {B}_{1,0}$ to $\mathfrak {B}_{1}$. Pure growth ($\mathbb {G}$) is defined in $\mathfrak {B}_{1,0}$, which maps ${\mathfrak {B}}_{1,0}$ to $\mathfrak {B}_\text {1,g}$, then an inelastic remodelling tensor $\mathbb {P}_\text {r}$ to another incompatible fictitious state $\mathfrak {B}_\text {1,gr}$. Finally, $\mathfrak {B}_\text {1,gr}$ can be assembled into $\mathfrak {B}_2$ by an elastic tensor $\mathbb {F}_\text {r}$, or further deforms into $\mathfrak {B}_\zeta$ under certain external loadings with a total elastic deformation $\mathbb {F}_\text {e}$. $\textbf{F}$ is the mapping from $\mathfrak {B}_1$ to $\mathfrak {B}_\zeta$ including two time-scale dynamics, a growth cycle described by $\textbf{F}_\text {eg}$ and cardiac dynamics within heartbeats described by $\textbf{F}_\zeta$

In this study, myocardial adaptation is modelled using the updated reference framework introduced in Guan et al. (2023b), as illustrated in Fig. 2 for one growth cycle from $\mathfrak {B}_1$ to $\mathfrak {B}_2$. Using the residual elastic deformation tensor $\textbf{F}_\text {r}$, the current unloaded configuration $\mathfrak {B}_1$ can be pulled back to ${\mathfrak {B}}_{0}$, which is a fictitious and incompatible state. This residual deformation tensor $\textbf{F}_\text {r}$ can account for the presence of non-zero residual stresses. Specifically, using polar decomposition, we have

8 $$\begin{aligned} \textbf{F}_\text {r}=\textbf{V}_\text {r} \boldsymbol{\textrm{R}}_\text {r}, \end{aligned}$$

with $\boldsymbol{\textrm{R}}_\text {r}$ a rotation tensor and $\textbf{V}_\text {r}$ a left-stretch tensor. The configuration ${\mathfrak {B}}_{0}$ can be transformed into ${\mathfrak {B}}_{1,0}$ under $\boldsymbol{\textrm{R}}_\text {r}$, another zero-stress incompatible configuration. The left-stretch tensor $\textbf{V}_\text {r}$ then can map $\mathfrak {B}_{1,0}$ to $\mathfrak {B}_{1}$.

Assuming that pure growth only occurs in a stress-free state (Göktepe et al. 2010), we then define a pure growth tensor $\mathbb {G}$ with respect to $\mathfrak {B}_{1,0}$, which leads to a grown and incompatible configuration ${\mathfrak {B}}_\text {1,g}$, further followed by a remodelling tensor $\mathbb {P}_r$ to another incompatible fictious state ${\mathfrak {B}}_\text {1,gr}$. Finally, $\mathfrak {B}_\text {1,gr}$ can be assembled into the unloaded configuration $\mathfrak {B}_2$ by an elastic tensor $\mathbb {F}_\text {r}$, or further deforms into $\mathfrak {B}_\zeta$ under certain external loadings with a total elastic deformation $\mathbb {F}_\text {e}$. The configuration mapping from $\mathfrak {B}_2$ to $\mathfrak {B}_\zeta$ is treated as a short-time scale dynamic problem, i.e. within heartbeats. Here we define a growth cycle as the configuration change from $\mathfrak {B}_1$ to $\mathfrak {B}_2$ with $\mathfrak {B}_2$ the reference configuration for the next growth cycle. With the help of $\mathbb {F}_\text {r}$ again, we can obtain ${\mathfrak {B}}_{2,0}$ similar to ${\mathfrak {B}}_{1,0}$. Finally, the total elastic tensor at $\mathfrak {B}_\zeta$ is given by

9 $$\begin{aligned} \mathbb {F}_\text {e}= \textbf{F} \textbf{V}_\text {r} \mathbb {G}^{-1}\mathbb {P}_\text {r}^{-1} \end{aligned}$$

with $\textbf{F}$ the mapping from $\mathfrak {B}_1$ to $\mathfrak {B}_2$, and the equivalent myocardial passive stress is

10 $$\begin{aligned} \boldsymbol{{\sigma }}^\text {p}=\mathbb {F}_\text {e} \frac{\partial {W^\text {p}}(\mathbb {F}_\text {e})}{\partial \mathbb {F}_\text {e}}-p\boldsymbol{\textrm{I}}, \end{aligned}$$

where $W^\text {p}$ is the strain energy density function defined in (1). We further assume $\mathbb {P}_\text {r} = \boldsymbol{\textrm{I}}$ for simplification in this study to focus on ventricular wall geometrical adaptation, and each growth cycle roughly corresponds to one week. For more details of this growth framework, please refer to Guan et al. (2023b). Similarly, the residual deformation from $\mathfrak {B}_\text {1,gr}$ to $\mathfrak {B}_2$ is given by

11 $$\begin{aligned} \mathbb {F}_\text {r}= \textbf{F}_\text {eg} \textbf{V}_\text {r} \mathbb {G}^{-1} \end{aligned}$$

with $\textbf{F}_\text {eg}$ is the mapping from $\mathfrak {B}_\text {1,gr}$ to $\mathfrak {B}_2$. The residual deformation $\mathbb {F}_\text {r}$ in $\mathfrak {B}_2$ is determined by solving the stress equilibrium equation under zero loading, which is considered to be a long-time scale for growth, see Algorithm 1. Please note that the geometries corresponding to $\mathfrak {B}_1$, $\mathfrak {B}_2$, and $\mathfrak {B}_\zeta$ are all experimentally or clinically observable.

**Algorithm 1:**
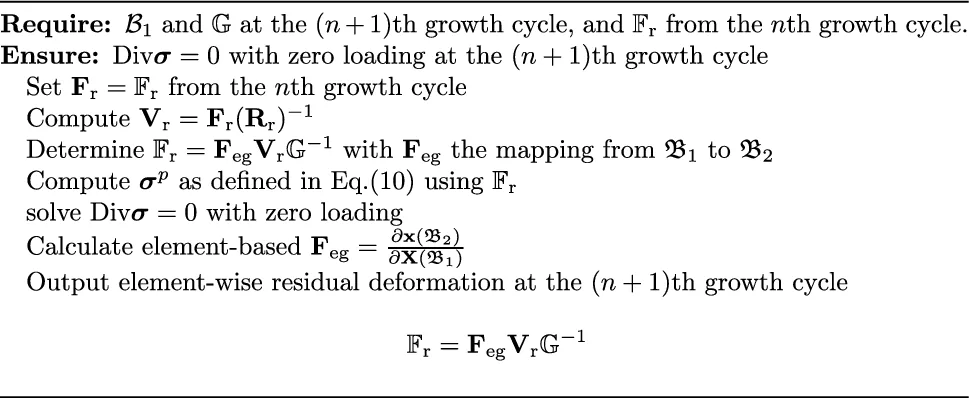
Residual deformation in $\mathfrak {B}_2$ after growth from $\mathfrak {B}_1$.

Myofibres are usually organized into a layered structure characterized by three mutually orthogonal directions, the so-called fibre-sheet-normal system (Gilbert et al. 2012; Ferreira et al. 2014). In a growth cycle, for example from $\mathfrak {B}_{1}$ to $\mathfrak {B}_{2}$ as shown in Fig. 2, the unit vectors $\textbf{f}_1$, $\boldsymbol{\textrm{s}}_1$, and $\boldsymbol{\textrm{n}}_1$ in $\mathfrak {B}_{1}$ will be deformed into

12 $$\begin{aligned} \textbf{f}^{'}=\textbf{F}_\text {eg} \textbf{f}_1, \qquad \boldsymbol{\textrm{s}}^{'}=\textbf{F}_\text {eg} \boldsymbol{\textrm{s}}_1, \qquad \boldsymbol{\textrm{n}}^{'}=\textbf{F}_\text {eg} \boldsymbol{\textrm{n}}_1. \end{aligned}$$

The vectors $\textbf{f}^{'}_1$, $\boldsymbol{\textrm{s}}^{'}_1$, and $\boldsymbol{\textrm{n}}^{'}_1$ are generally not orthogonal. When $\mathfrak {B}_{2}$ is adopted as the updated reference configuration for the subsequent growth cycle, we implicitly assume an additional remodelling process to re-establish an orthogonal fibre-sheet-normal system ($\textbf{f}^u - \boldsymbol{\textrm{s}}^u - \boldsymbol{\textrm{n}}^u$), such that

13 $$\begin{aligned} \textbf{f}^{u}=\frac{\textbf{f}^{'}}{|\textbf{f}^{'}|}, \quad \boldsymbol{\textrm{n}}^{u}=\frac{\textbf{f}^{u} \times \boldsymbol{\textrm{s}}^{'}}{|\textbf{f}^{u} \times \boldsymbol{\textrm{s}}^{'}|}, \quad \boldsymbol{\textrm{s}}^{u}=\frac{\boldsymbol{\textrm{n}}^{u} \times \textbf{f}^{u}}{|\boldsymbol{\textrm{n}}^{u} \times \textbf{f}^{u}|}. \end{aligned}$$

Further discussion on this re-orthogonalisation proccess an be found in Sect. 4.

The adopted growth framework is based on the kinematic growth theory (Rodriguez et al. 1994). Instead of using a fixed reference configuration as in most kinematic growth cardiac models (Göktepe et al. 2010), an updated reference is used for each growth cycle, and it further incorporates growth-induced residual stresses. It does not model each individual constituent’s growth, deposition, removal, and stress-free configuration as in the constrained mixture theory (Humphrey and Rajagopal 2002). Kinematic growth theory has been widely used to capture the geometric and functional adaptation of the ventricle at the organ scale (Göktepe et al. 2010). As the aim of this study is to investigate organ-level RV adaptation under volume overload, the kinematic growth approach provides a computationally feasible way of description. Constituent-specific remodelling could be achieved within this adopted updated reference approach built upon the constrained mixture theory, as demonstrated in our previous work on fibrosis following myocardial infarction (Guan et al. 2025).

### Growth laws

In this study, we will mainly focus on eccentric growth in the RV driven by stretch and stress due to regurgitation induced volume overload in the RV, then briefly explore the combination of eccentric and concentric growth (hybrid), and finally allow LV growth simultaneously.

By assuming eccentric growth along the unit myofibre direction ($\bar{\textbf{f}}$) driven by fibre stretch, and concentric growth along its unit cross-fibre direction ($\bar{{\mathbf{c}}}$) transmurally driven by peak systolic active stress, we define a general growth tensor as

14 $$\begin{aligned} \mathbb {G}=\vartheta ^{\text {f}}\,\bar{\textbf{f}}\otimes \bar{\textbf{f}}\,+\vartheta ^{\text {c}}\,\bar{{\mathbf{c}}}\otimes \bar{{\mathbf{c}}}\,+\bar{{\mathbf{l}}}\otimes \bar{{\mathbf{l}}}, \end{aligned}$$

where $\vartheta ^{\text {f}}$ and $\vartheta ^{\text {c}}$ are the incremental growth ratios respectively along the myofibre ($\bar{\textbf{f}}$) and cross-myofibre ($\bar{\boldsymbol{\textrm{c}}}$) directions with respect to the updated reference configuration, and $\bar{\boldsymbol{\textrm{l}}}$ is the orthogonal direction to both $\bar{\textbf{f}}$ and $\bar{\boldsymbol{\textrm{c}}}$. The reason for considering growth along $\boldsymbol{\textrm{c}}$ rather than $\boldsymbol{\textrm{l}}$ is that $\boldsymbol{\textrm{c}}$ is along the transmural direction, and thus directly related to wall thickening or thinning. Similar growth tensor definitions have also been used in Rausch et al. (2011), when modelling myocardial concentric growth. It should be noted that the exact form of this growth tensor for myocardial hypertrophy remains unknown.

In eccentric growth, assuming growth only occurs along the myofibre direction, thus (14) is rewritten as

15 $$\begin{aligned} \mathbb {G}=\vartheta ^{\text {f}}\,\bar{\textbf{f}}\otimes \bar{\textbf{f}}\,+\bar{{\mathbf{c}}}\otimes \bar{{\mathbf{c}}}\,+\bar{{\mathbf{l}}}\otimes \bar{{\mathbf{l}}}. \end{aligned}$$

Similarly, concentric growth can be achieved by setting $\vartheta ^{\text {f}}=1$ while retaining $\vartheta ^{\text {c}}$. When both $\vartheta ^{\text {f}}$ and $\vartheta ^{\text {c}}$ are different from 1, then it describes a hybrid growth.

To determine growth from $\mathfrak {B}_{1}$ to $\mathfrak {B}_{2}$, we need to find out the myofibre direction $\bar{\textbf{f}}$, which is obtained by pulling the orthogonal fibre-sheet-normal system $\textbf{f}_1 -\boldsymbol{\textrm{s}}_1-\boldsymbol{\textrm{n}}_1$ at $\mathfrak {B}_{1}$ back to $\mathfrak {B}_{1,0}$ using the residual stretch tensor $\textbf{V}_\text {r}$, such that

16 $$\begin{aligned} \overline{{\mathbf{f}}} = & \,\frac{{({\mathbf{V}}_{{\mathrm{r}}} )^{{ - 1}} {\mathbf{f}}_{1} }}{{|({\mathbf{V}}_{{\mathrm{r}}} )^{{ - 1}} {\mathbf{f}}_{1} |}},\,{\mathbf{s}}_{{1,0}} = \frac{{({\mathbf{V}}_{{\mathrm{r}}} )^{{ - 1}} {\mathbf{s}}_{1} }}{{|({\mathbf{V}}_{{\mathrm{r}}} )^{{ - 1}} {\mathbf{s}}_{1} |}}, \\ & \,{\mathrm{n}}_{{1,0}} = \frac{{({\mathbf{V}}_{{\mathrm{r}}} )^{{ - 1}} {\mathrm{n}}_{1} }}{{|({\mathbf{V}}_{{\mathrm{r}}} )^{{ - 1}} {\mathrm{n}}_{1} |}}. \\ \end{aligned}$$

Then, the cross-myofibre directions are defined as

17 $$\begin{aligned} \bar{{\mathbf{l}}}=\frac{\bar{\textbf{f}} \times {\mathbf{s}}_{1,0}}{|\bar{\textbf{f}} \times {\mathbf{s}}_{1,0}|}, \quad \bar{{\mathbf{c}}}=\frac{\bar{{\mathbf{l}}} \times \bar{\textbf{f}}}{|\bar{{\mathbf{l}}} \times \bar{\textbf{f}}|}, \end{aligned}$$

in which $\bar{\boldsymbol{\textrm{c}}}$ is along the radial (transmural) direction in general. The growth tensors defined in (14) and (15) are phenomenological to capture the elongation of myofibres and the wall thickening. The actual growth tensor could be more complex.

We adopt the evolution equation for stretch-driven fibre growth ($\theta ^\text {f}$) from Göktepe et al. (2010), and it is defined with respect to the initial non-grown state,

18 $$\begin{aligned}&\dot{\theta ^\text {f}}_{n+1}=\frac{1}{\tau ^\text {f}} \left( \frac{\theta ^\text {max,f} - \theta ^\text {f}_{n+1}}{\theta ^\text {max,f} - 1} \right) ^{\gamma ^\text {f}} \mathcal {H}(\lambda ^e - \lambda ^\text {crit}) \quad \text {with} \nonumber \\&\quad \lambda ^e=| \mathbb {F}_e\cdot \bar{\mathbf{f}} |, \end{aligned}$$

in which $\gamma ^\text {f}$ and $\tau ^\text {f}$ are constants, $\theta ^\text {max,f}$ is the maximum growth ratio along the fibre direction, $\lambda ^\text {crit}$ is the critical threshold of fibre stretch, $\mathbb {F}_e$ is the total elastic stretch tensor, and $\mathcal {H}(\cdot )$ is the Heaviside function. The incremental growth ratio from the *n*th growth cycle to the (n+1)th cycle is defined as

19 $$\begin{aligned} \vartheta ^\text {f}_{n+1}=\theta ^\text {f}_{n+1}/\theta ^\text {f}_{n}. \end{aligned}$$

In a similar way, the evolution equation for stress-driven cross fibre growth $(\theta ^\text {c})$ is given by

20 $$\begin{aligned} \dot{\theta ^\text {c}}_{n+1}=\frac{1}{\tau ^\text {c}} \left( \frac{\theta ^\text {max,c} - \theta ^\text {c}_{n+1}}{\theta ^\text {max,c} - 1} \right) ^{\gamma ^\text {c}} \mathcal {H}(\text {tr}(\boldsymbol{{\sigma }}^a) - p^\text {crit}), \end{aligned}$$

where $\gamma ^\text {c}$ and $\tau ^\text {c}$ are constants, $\theta ^\text {max,c}$ is the maximum growth ratio along the cross-fibre direction, $p^\text {crit}$ is the critical threshold of active tension. Accordingly, the incremental growth ratio along the cross-fibre direction from the *n*th growth cycle to the (n+1)th cycle is given by

21 $$\begin{aligned} \vartheta ^\text {c}_{n+1}=\theta ^\text {c}_{n+1}/\theta ^\text {c}_{n}. \end{aligned}$$

Equations (18) and (20) are discretized using the finite difference method for the first-order material time derivative. In each growth cycle, we allow $\theta ^\text {f}$ and $\theta ^\text {c}$ to reach their maximum possible growth. The precise units for $\tau ^\text {f}$ and $\tau ^\text {c}$ could be determined from experimental data, such as days or weeks. The simulation results from the baseline case without regurgitation are used to determine $\lambda ^\text {crit}$ in (18): the spatial mean myofibre stretch at end-diastole, and $p^\text {crit}$ in (20): the spatial mean of the maximum of the trace of active stress tensor $\boldsymbol{{\sigma }}^a$ in systole. Specifically,

22 $$\begin{aligned} \lambda ^\text {crit}=\frac{1}{N}\sum _{i=1}^N \lambda _{i}^\text {max}, \quad \text {and} \quad p^\text {crit}=\frac{1}{N}\sum _{i=1}^N\,p_i^\text {max}, \end{aligned}$$

where *N* is the total number of elements of the biventricular model, $\lambda _i^\text {max}$ is the maximum myofibre stretch in diastole for the *i*th element, and $p_i^\text {max}$ is the maximum of the trace of active stress $\text {tr}(\boldsymbol{{\sigma }}^a)$ in systole.

The reason for choosing the trace of $\boldsymbol{{\sigma }}^a$ is motivated by the fact that, during systole, active tension in the myofibres dominates the total stress, typically reaching values on the order of 100 kPa (Mangion et al. 2018), while passive contributions are relatively small due to reduced fibre stretch or shortening compared to the end-diastolic state. From a physiological perspective, increased contractile demand, often mediated through calcium handling and active force generation, has been associated with hypertrophic responses (Wilkins and Molkentin 2004), thus provides a suitable stimulus for concentric growth. It is worthwhile to mention that future work shall investigate other types of triggers, such as the total stress tensor over cardiac cycles. See Witzenburg and Holmes (2017) for a comparative study on various growth laws and triggers.

### Model summary

Simulated cases are listed below, and relevant parameters are included in Table 1.

- the **baseline** case: Before modelling RV regurgitation and the induced growth, we need to simulate the baseline case without any regurgitation in the RV. Note LV and RV material properties shall be different. We assume the septum and LVFW have the same material properties (both passive and active), but different from the RVFW. We firstly inversely estimated passive parameters in (1) to match in vivo measured end-diastolic volumes of the LV and RV following a multistep approach (Gao et al. 2017). During diastole, the LV is preloaded with a linearly increased pressure until it reaches a population-based normal end-diastolic pressure. Here, we set 6.6 mmHg, similar to the RV that is preloaded with a pressure 2.0 mmHg. The active contractile parameters $K_a^p$ for LV and RV are adjusted to match the measured ejection fraction. Other parameters of this bi-ventricular model are adapted from Guan et al. (2022a) to ensure blood pressures are within the physiological ranges at different compartments for a healthy human heart. For the baseline case, the regurgitant resistances of the pulmonary valve and tricuspid valve are set to be sufficiently large, as can be seen in Table 1.
- the **PR** case (acute): Based on the baseline case, only the PV regurgitant resistance is set to be 0.1% of the baseline value.
- the **TR** case (acute): Based on the baseline case, only the TV regurgitant resistance is set to be 0.1% of the baseline value.
- the **P&TR** case (acute): Based on the baseline case, while both the PV and TV regurgitant resistances are set to be 0.1% of the baseline values.
- the **PR-Ecc** case: Eccentric growth in the RV, including both the RVFW and septum, is considered, starting from the acute PR case
- the **TR-Ecc** case: Eccentric growth in the RV, including both the RVFW and septum, is considered, starting from the acute TR case.
- the **P&TR-Ecc** case: Eccentric growth in the RV, including both the RVFW and septum, is considered, starting from the acute P&TR case.
- the **hybrid-RV** case: Both eccentric and concentric growth in the RV, including both the RVFW and septum, is studied, starting from the acute P&TR case.
- the **hybrid-Biv** case: In this case, we allow the LV growth simultaneously as the RV growth by allowing both eccentric and concentric growth. This is a preliminary study to explore the interdependence between the left and right hearts in a simplified growth scenario.

From the baseline case, we obtain $\lambda ^\text {crit}$ using the volumetric averaged myofibre stretch at end diastole, and similarly for $p^\text {crit}$, the spatial mean of the maximum of $\text {tr}(\boldsymbol{{\sigma }}^a)$ during systole. For RV growth cases, the termination criterion for growth is when the incremental growth ratio between two consecutive growth cycles is less than 1%. We found that PR-/TR-/P&TR-Ecc cases can finish in 7 growth cycles. For a like-for-like comparison, the same number of growth cycles is also applied to the hybrid-RV and hybrid-Biv cases.

**Table 1:** Parameter values for all studied cases

Myocardial material parameters Guan et al. (2022b)									
	*a* (kPa)	*b*	$a_\text {f}$ (kPa)	$b_\text {f}$	$a_\text {n}$ (kPa)	$b_\text {n}$	$a_\text {fs}$ (kPa)	$b_\text {fs}$	$K_a^p$
LV	0.1113	2.1561	1.7257	5.6326	0.3566	2.2903	0.1000	0.1534	0.22
RV	0.1833	2.1561	2.8428	5.6326	0.5875	2.2903	0.1647	0.1534	0.35
Parameters of eccentric and concentric growth laws Guan et al. (2023b); Göktepe et al. (2010)									
$\tau ^\text {f}$	$\gamma ^\text {f}$	$\tau ^\text {c}$	$\gamma ^\text {c}$	$\lambda ^\text {crit}$	$p^\text {crit}$ (MPa)	$\theta ^\text {f,max}$	$\theta ^\text {c,max}$		
0.2	1.0	0.4	1.0	1.13	0.05	1.4	2.0		
Parameters of the lumped circulation model Guan et al. (2020)									
$R^\text {AV}$	$R^\text {MV}$	$R^\text {TV}$	$R^\text {PV}$	$R^\text {Sys}$	$R^\text {Pul}$	($\text {MPa}\cdot \text {mm}^2 \cdot \text {s}/ \text {tonne}$)			
1.5	1.0	1.0	1.2	25	40				
$C_\text {LA}$	$C_\text {RA}$	$C_\text {PA}$	$C_\text {Ao}$	($\text {N}/\text {mm}$ )					
0.05	0.05	0.15	1.0						
Parameters for regurgitation ($\text {MPa}\cdot \text {mm}^2 \cdot \text {s}/ \text {tonne}$)									
	$R^\text {PR}$	$R^\text {TR}$							
Baseline	10,000	10,000							
PR	10	10,000							
TR	10,000	10							
P&TR	10	10							

## Results

### Acute cases

**Fig. 3:**
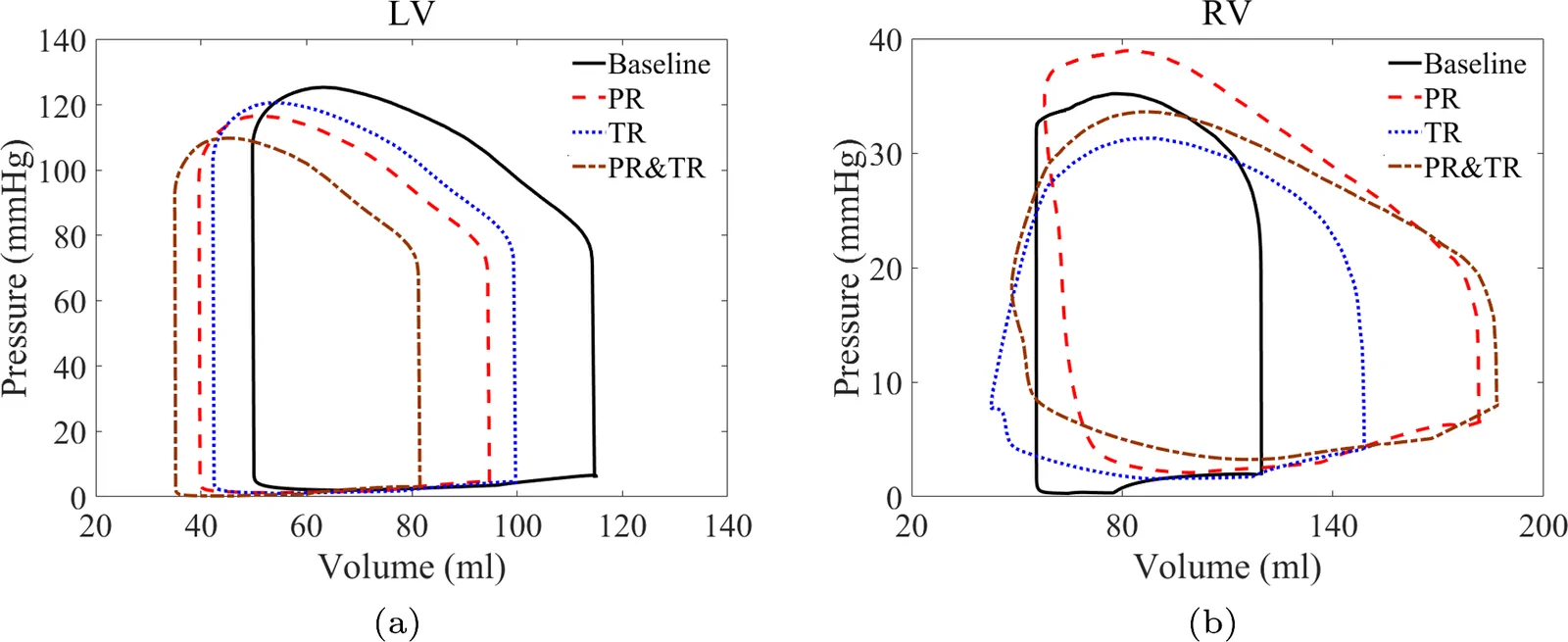
Instantaneous pressure-volume loops of the LV **a** and the RV **b** from the baseline case, with PR, TR and both PR and TR. Note, G&R has not started

The pressure-volume (P-V) loops of the LV and RV for the baseline and three regurgitant cases are shown in Fig. 3. As can be seen from Fig. 3a, RV regurgitation actually impairs LV filling and shifts the LV P-V loops leftward. For RV hemodynamics, the PR case experiences a regurgitant volume of 72 mL (58.1% of RV stroke volume (SV)), the TR case experiences a regurgitant volume of 51 mL (47.2%), and the P&TR case has a regurgitant ratio of 37.1% from the PV and 44.3% from the TV. All cases experience severe regurgitation in the RV. Stroke work in the RV is significantly higher in regurgitant cases, particularly with PV regurgitation, as seen in Fig. 3b. Both PR and P&TR cases result in nearly doubled RV end-diastolic volumes (EDVs) compared to the baseline case. The TR case has a slightly lower peak systolic pressure but a reduced end-systolic volume (ESV) due to decreased afterload, whereas the PR case shows a higher peak systolic pressure due to increased preload. In the P&TR case, the RV reaches the largest EDV, indicating excessive passive stretch in diastole.

Haemodynamic results are further summarised in Table 2, and relevant ranges from clinical literature. While the standard ejection fraction, defined as SV/EDV, has limitations to assess ventricular pump function in the presence of regurgitation, its value may appear normal, though the forward blood flow entering the pulmonary circulation is reduced. A more informative measure is the effective ejection fraction (eEF), computed using the forward blood volume into the pulmonary circulation rather than SV (Zhang et al. 2026). As can be seen from Table 2, eEF for all three regurgitant cases is much lower compared to EF, with the P&TR case value even below the critical value (25%) identified by Zhang et al. (2026). PR and TR severities fall within ranges reported for moderate and severe cases. Significantly increased RV cavity volume can be found in the presence of regurgitation compared to the baseline case, while remaining within the reported clinical ranges in the literature. The highest RV cavity volume in the P&TR case is in line with clinical observations that patients with combined PR and TR may be at higher risk of RV failure due to excessive volume overload (Śpiewak et al. 2020). In general, all cases are broadly consistent with clinical observations in terms of magnitude and trends.

**Table 2:** Haemodynamic results for the baseline and regurgitation cases, with reported clinical ranges from the literature for comparison

Condition	Metric	RV	RV clinical data	LV	LV clinical data
Baseline	EDP (mmHg)	2.0	1–7 Haleem et al. (2025)	6.6	5–12 Fukuta and Little (2008)
	ESP (mmHg)	35	15–30 Haleem et al. (2025)	125	90–140 Fukuta and Little (2008)
	EDV (ml)	120	Male: 124–248; Female: 85–168 Petersen et al. (2016)	115	Male:109–218; Female: 88–161Petersen et al. (2016)
	ESV (ml)	55	Male: 47–123; Female: 27–77 Petersen et al. (2016)	50	Male: 39–97; Female: 31–68 Petersen et al. (2016)
	SV (ml)	65	Male: 62–131; Female: 48–99 Petersen et al. (2016)	65	Male: 59–132; Female: 49–100 Petersen et al. (2016)
	PRV (ml)	0		∼	
	TRV (ml)	0		∼	
	PRF (%)	0		∼	
	TRF (%)	0		∼	
	EF (%)	54.2	Male: 45–65; Female: 47–68 Petersen et al. (2016)	56.5	Male: 48–69; Female: 51–70 Petersen et al. (2016)
	eEF(%)	∼		∼	
PR	EDP (mmHg)	6.5	11–17 Egbe et al. (2019)	4.9	*see Baseline*
	ESP (mmHg)	39	46±6 Egbe et al. (2019)	116	*see Baseline*
	EDV (ml)	182	136±66 Egbe et al. (2019)	94	69–206 Leonardi et al. (2022)
	ESV (ml)	58	117±18Egbe et al. (2019)	41	27–86 Leonardi et al. (2022)
	SV (ml)	124	117±13Egbe et al. (2019)	53	42-−108.3 Leonardi et al. (2022)
	PRV (ml)	72	48±12Egbe et al. (2019)	∼	
	TRV (ml)	0		∼	
	PRF (%)	58.1	27–60 Leonardi et al. (2022)	∼	
	TRF (%)	0		∼	
	EF (%)	68.1	55.0 Leonardi et al. (2022), 48±11 Egbe et al. (2019)	56.4	57.0 Leonardi et al. (2022), 53±9 Egbe et al. (2019)
	eEF(%)	28.6	<25 criticalZhang et al. (2026)	∼	
TR	EDP (mmHg)	4.2	6–14 Kim et al. (2025)	4.7	*see Baseline*
	ESP (mmHg)	31	28.5–43 Kim et al. (2025)	121	*see Baseline*
	EDV (ml)	150	114–194 (moderate TR)Caravita et al. (2025)	99	71–112(severe TR)Caravita et al. (2025)
	ESV (ml)	42	37–93(moderate TR) Caravita et al. (2025)	42	26–54(severe TR)Caravita et al. (2025)
	SV (ml)	108	32–61 Kim et al. (2025)	57	
	PRV (ml)	0		∼	
	TRV (ml)	51	45–62 (severe TR) Caravita et al. (2025)	∼	
	PRF (%)	0		∼	
	TRF (%)	47.2	53–67 (severe) Caravita et al. (2025)	∼	
	EF (%)	72.0	48–66 Caravita et al. (2025)	57.6	54–72 Caravita et al. (2025)
	eEF(%)	38	<25 critical Zhang et al. (2026)	∼	
P&TR	EDP (mmHg)	8.0	11–17 Egbe et al. (2019)	2.9	*see Baseline*
	ESP (mmHg)	34	46±6 Egbe et al. (2019)	110	*see Baseline*
	EDV (ml)	188	271±84 Śpiewak et al. (2020)	80	152±26 Śpiewak et al. (2020)
	ESV (ml)	48	146±74 Śpiewak et al. (2020)	35	70±22 Śpiewak et al. (2020)
	SV (ml)	140	76.2±17.4 Śpiewak et al. (2020)	45	48.9±8.4 Śpiewak et al. (2020)
	PRV (ml)	52	39.7±23.0 Śpiewak et al. (2020)	∼	
	TRV (ml)	62	≥45 Zhan et al. (2020)	∼	
	PRF (%)	37.1	27.4±15.1 Śpiewak et al. (2020)	∼	
	TRF (%)	44.3	≥50 Zhan et al. (2020)	∼	
	EF (%)	74.5	48.1±11.1 Śpiewak et al. (2020)	56.2	56.6±8.3 Śpiewak et al. (2020)
	eEF(%)	23.9	<25 critical Zhang et al. (2026)	∼	

EDP: end-diastolic pressure; ESP: end-systolic pressure; EDV: end-diastolic volume; ESV: end-systolic volume; SV: stroke volume; PRV: pulmonary regurgitation volume; TRV: tricuspid regurgitation volume; PRF: pulmonary regurgitation fraction; TRF: tricuspid regurgitation fraction; EF: ejection fraction; eEF: effective ejection fraction. Indexed clinical volumes, reported in $\mathrm{ml/m}^2$, are included in the corresponding volume metric cells by multiplying the average body surface area ($\mathrm{1.7m}^2$)

**Fig. 4:**
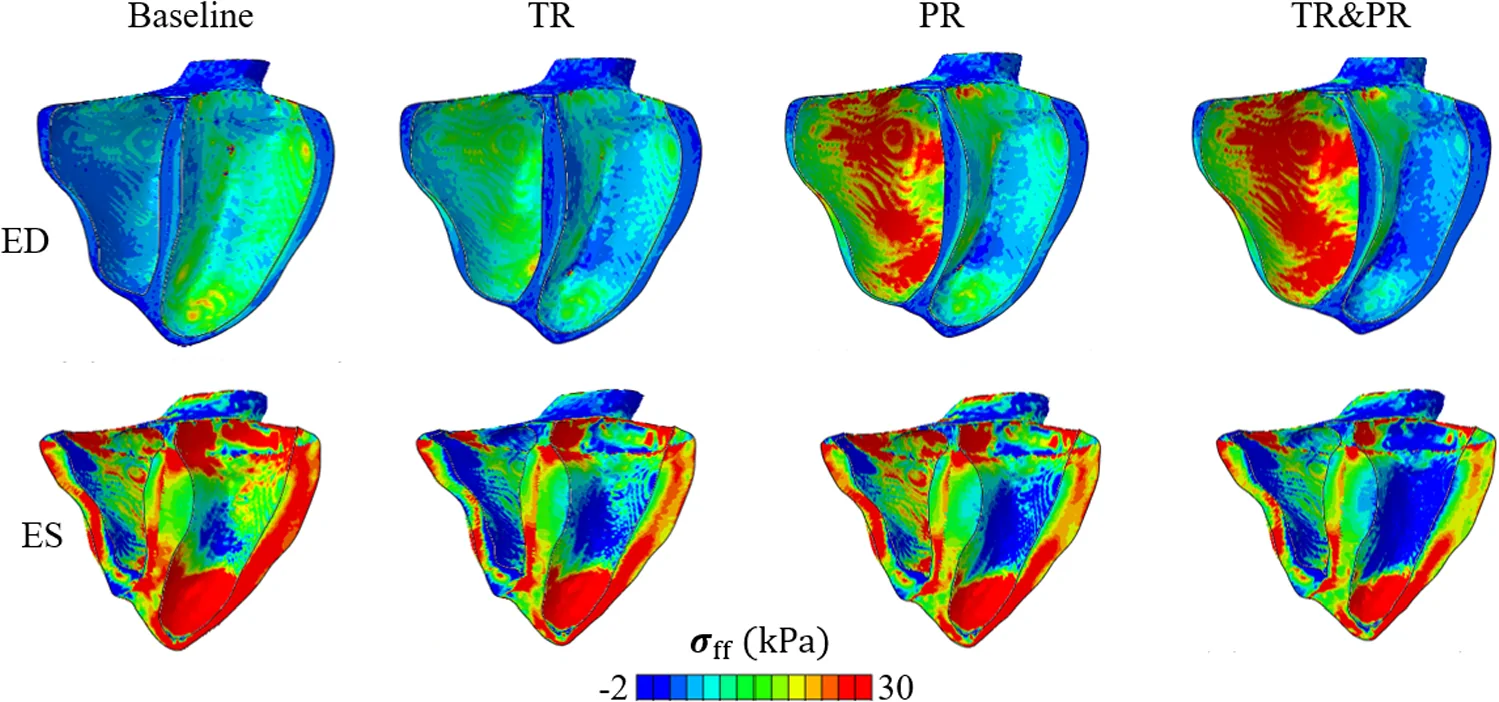
Distribution of myofibre stress ($\sigma _\text {ff}$) at end diastole (ED) and end systole (ES) before any G&R with deformed biventricle geometries

Distributions of myofibre stress at end-diastole and end-systole can be found in Fig. 4. Compared to the baseline case, the TR case experiences a slightly higher level of stress at end-diastole, with the septum being pushed towards LV, and much higher levels can be found for both the PR and P&TR cases, with the septum being pushed further towards LV due to PR in diastole. Interestingly, the systolic stress for the TR case is lower compared to the baseline case, which could be partially explained by the reduced afterload caused by TR in systole. Similar results can be found for the P&TR case.

### Eccentric growth in the RV

In this section, we will summarise the results of RV eccentric growth due to either PR or TR or both, but no growth in the LVFW. Note, we assume the septum experiences the same growth as the RVFW.

**Fig. 5:**
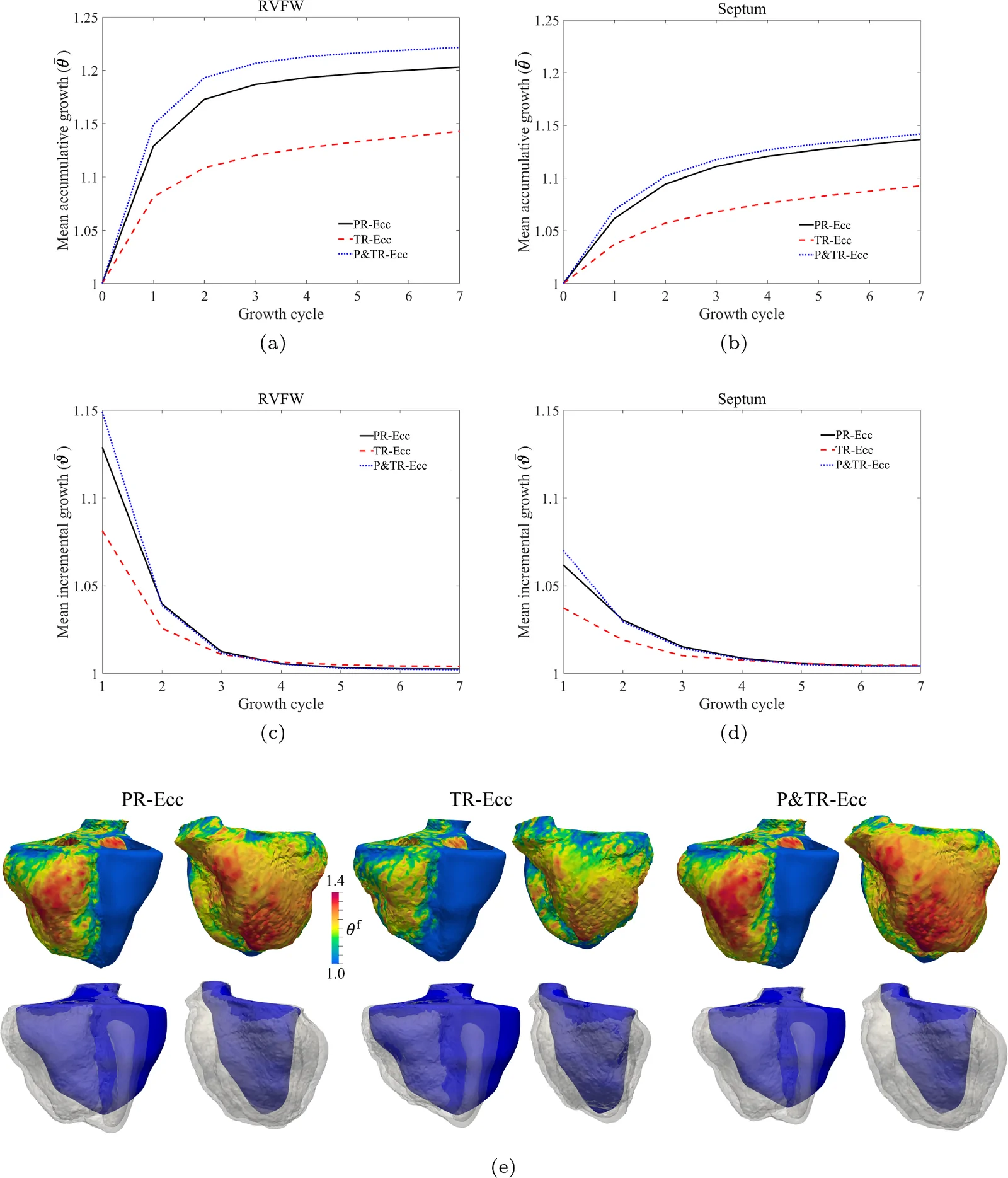
G&R results of the PR, TR and P&TR eccentric growth cases, specifically the mean accumulative growth ratio in the RVFW (**a**) and the septum (**b**), with corresponding the mean incremental growth ratios (**c**) and (**d**). The distribution of ${\theta }$ in the grown heart (**e**) from different views in the top panel, and the comparison of geometry before (blue) and after (grey) growth in the bottom panel

Mean accumulative growth ratios ($\bar{\theta }$) of eccentric growth in the RVFW are shown in Fig. 5a, in which the P&TR-Ecc case achieves the largest growth amount with $\bar{\theta }=1.22$ at the end of 7$^\text {th}$ growth cycle compared to the baseline geometry. The PR-Ecc case reaches $\bar{\theta }=1.20$ in the end, and the least growth, $\bar{\theta }=1.14$, can be found for the TR-Ecc case. A similar trend can be found in the septum but with less $\bar{\theta }$ compared to the RVFW, shown in Fig. 5b. The different growth pattern between the RVFW and septum could be due to (1) different loading conditions on the septum that experiences both LV and RV pressures, (2) different mechanical properties, and (3) a much thicker wall for the septum. Figure 5c shows the mean incremental growth ($\bar{\vartheta }$) approaching one as the RVFW grows. Again, a similar trend can be found for the septum in Fig. 5d.

The top panel of Fig. 5e shows the distribution of ${\theta ^\text {f}}$ in the grown heart at the end of growth (the 7$^\text {th}$ cycle). It can be found that the RVFW growth mainly occurs at the anterior and lateral RVFW (i.e. below RVOT), with a large region of the inferior RVFW experiencing a high level of growth. The growth amount is much less for the TR-Ecc case compared to the PR-Ecc case, as it might be expected since TR causes volume overload in the right atrium, leading to an increased preload for the RV, while PR directly overloads the RV in diastole. Under both PR and TR, the RV experiences the highest growth because both PR and TR can trigger RV growth. We further overlap the grown geometry with the baseline geometry under unloaded conditions in the bottom panel of Fig. 5e. It can be found that the RV shows a significant dilation with longitudinal elongation, especially in the PR-Ecc and P&TR-Ecc cases. Following RV growth, it distorts the LV shape, such as longitudinal stretch caused by longitudinal elongation in the RVFW and septum. We will further explore in Sect. 3.4 how such geometrical distortion caused by RV growth affects LV structural and functional adaptation if LV growth is also allowed.

**Fig. 6:**
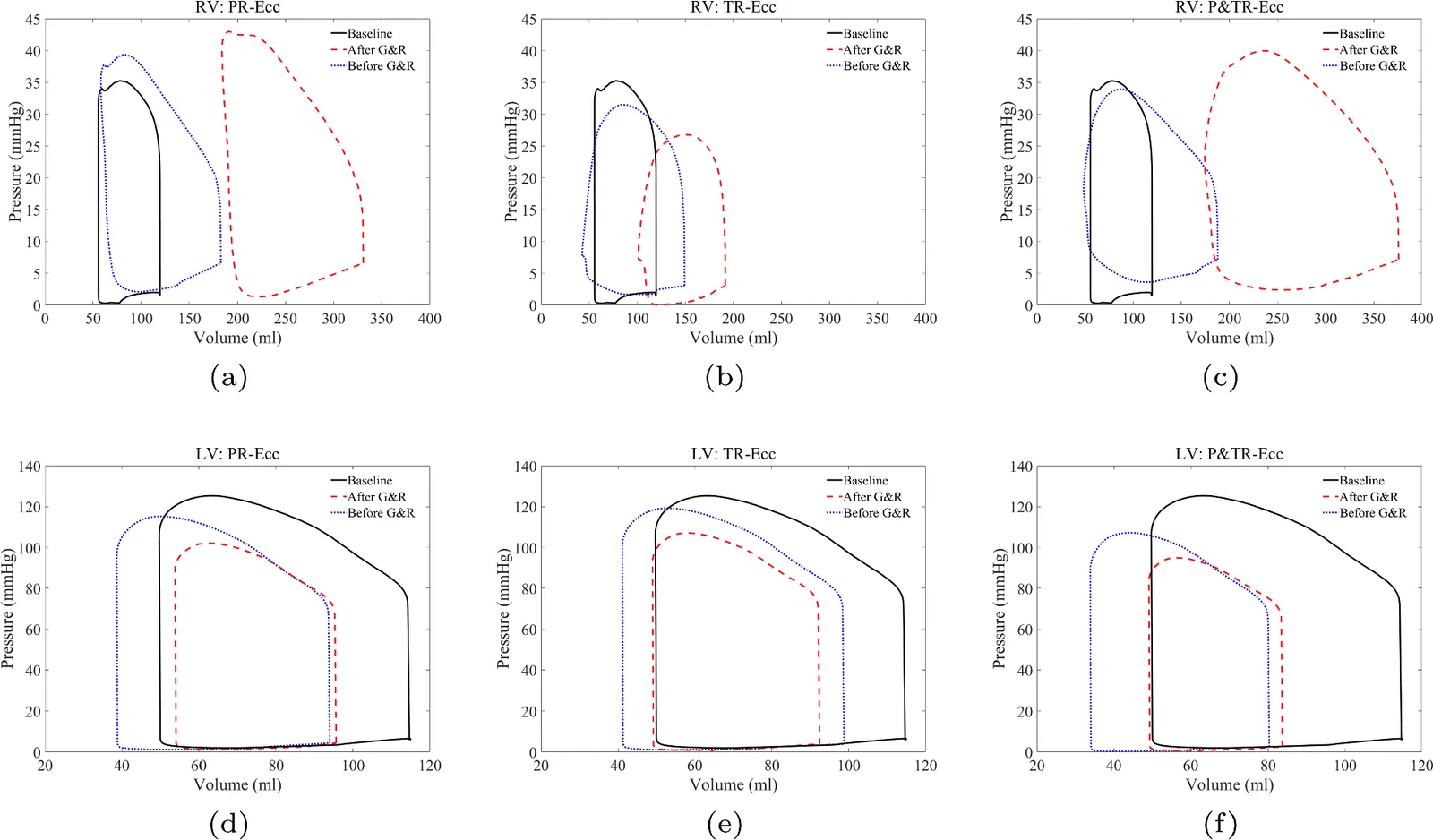
Comparison of the instantaneous pressure-volume loops in the baseline, the regurgitation state before and after growth in the RV for PR (**a**), TR (**b**) and P&TR (**c**) cases, and corresponding LV loops in (**d**–**f**)

Figure 6 compares P-V loops of the baseline, the acute regurgitant and the final grown states. The RV P-V loops are shifted rightward with much increased EDV and ESV as shown in Figs. 6a–c. In particular, for the cases with PR, their RV EDVs are almost three to four times larger than the baseline case. The most dilation occurs in the P&TR case with the largest RV EDV, nearly 370 mL. There is also a minor increase in end-systolic pressure at the final grown state compared to the acute state for the PR-Ecc and P&TR-Ecc cases, but reduced for the TR-Ecc case due to the reduced afterload caused by TR. The rightward shift of the P-V loops indicates increased volume loading, which promotes RV dilation as a compensatory mechanism to preserve stroke volume. With sustained overload, this adaptive response may become maladaptive, leading to progressive RV dilation and eventual functional deterioration, particularly in the P&TR case. The predicted P-V loop characteristics are in line with clinical observations in severe PR (Egbe et al. 2019) and TR (Tadic et al. 2022). Figure 6d–f show the LV P-V loops. Although there is no growth in LV, RV cavity enlargement, septum structural change, affects LV dynamics. Compared to the baseline case, all LV P-V loops are shifted leftward with decreased EDV and ESV, a phenomenon known as ventricular interdependence (Galloo and Unger 2026). Interestingly, RV growth further hinders LV contraction in systole with larger ESV, thus reducing SV and lowering peak systolic pressure from the acute state to the final grown state. For example, the stroke volume in the acute P&TR case is 45 mL, but reduced to 30 mL with an ejection fraction of 35% at the final grown state. This may suggest that LV also needs to adapt its structure and function to meet the demand for blood supply to the whole body. Otherwise, it would lead to a bi-ventricular failure.

### Hybrid growth in the RV

**Fig. 7:**
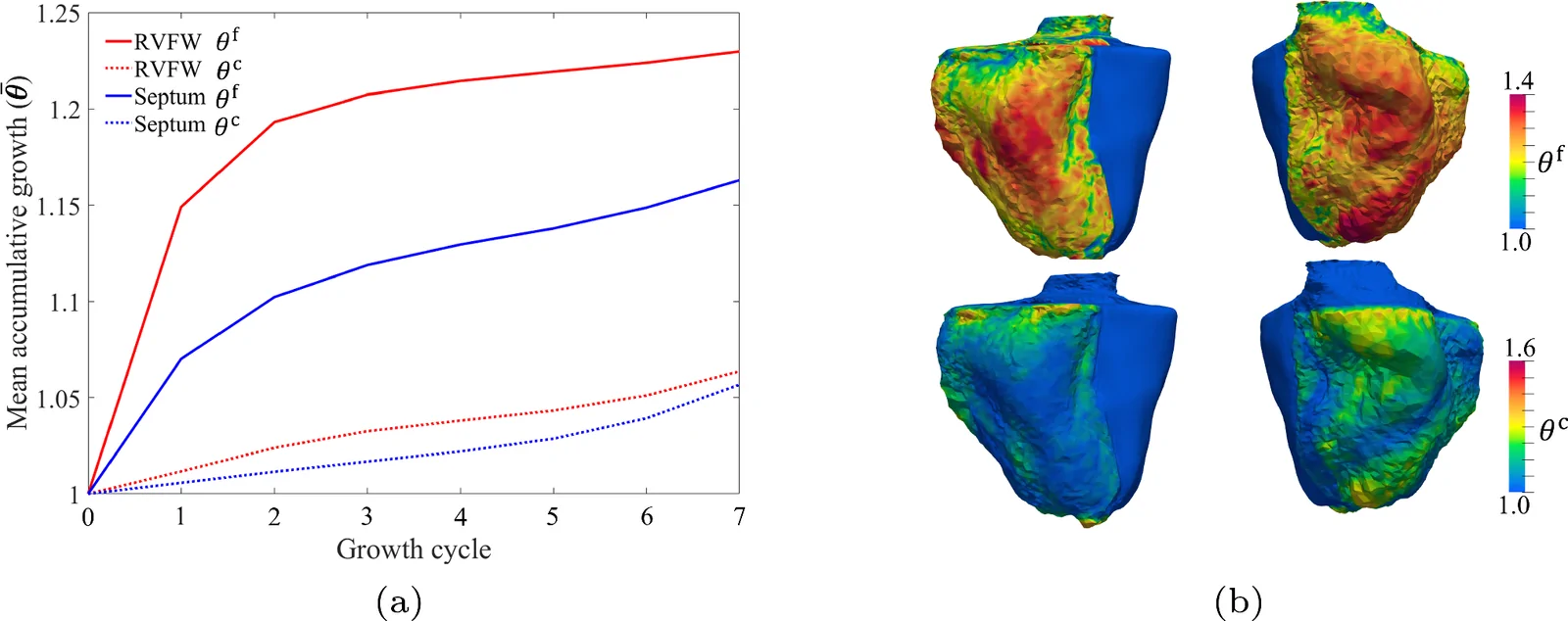
Hybrid G&R in the RV. The increased mean accumulative growth ratio in the RVFW and the septum (**a**), along with the distributions of the eccentric growth ratio ($\theta ^\text {f}$) and the concentric growth ratio ($\theta ^\text {c}$) at the end of the growth cycle (**b**)

It can be found in Fig. 7a that the mean eccentric growth ratio in the RVFW can reach 1.23 at the 7$^\text {th}$ growth cycle, which is much larger than that of the septum, about $\bar{\theta ^\text {f}}=1.16$. The mean concentric growth ratios for the RVFW and septum are almost the same at the 7$^\text {th}$ growth cycle, about $\bar{\theta ^\text {c}}=1.06$, much less compared to the concentric growth. Distributions of $\theta ^\text {f}$ and $\theta ^\text {c}$ at the 7$^\text {th}$ growth cycle are shown in Fig. 7b, where high levels of $\theta ^\text {f}$ throughout the RV can be found that is very similar to the P&TR-Ecc case as shown in Fig. 5e, with a low level of concentric growth in general though the apex and ventricular base seem growing more, which might be due to the geometrical singularity in apex and boundary condition effects near the base.

**Fig. 8:**
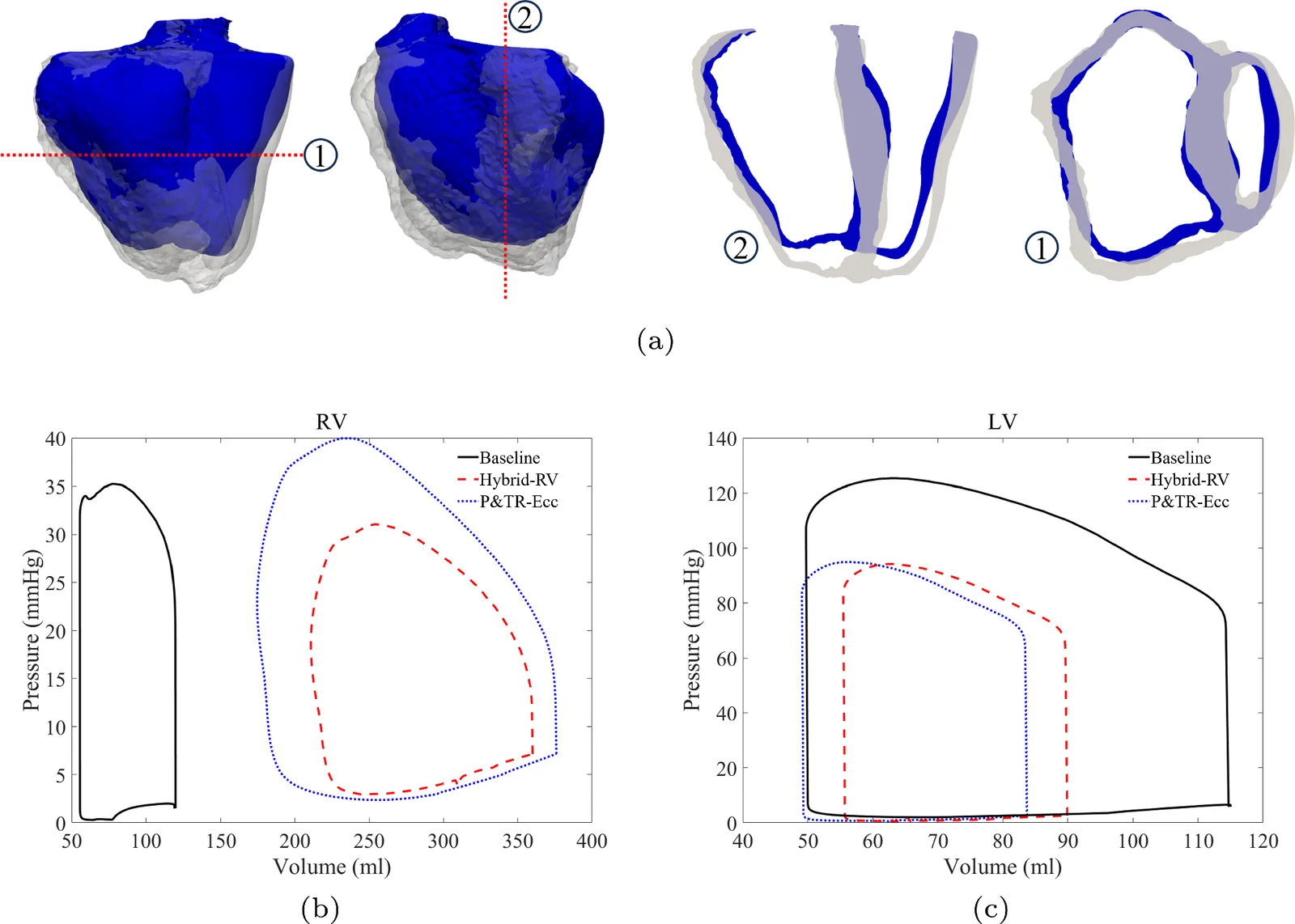
Comparison of RV G&R with concentric growth (grey) and without (blue) $\theta ^\text {c}$ (**a**), along with the cut views from positions (red dotted lines), and instantaneous pressure-volume loops in the RV (**b**) and the LV (**c**)

Figure 8a further compares the final grown geometry of this hybrid case to the P&TR-Ecc case (coloured by blue). Hybrid growth in the RV leads to a different configuration, especially the further longitudinal elongation near the apex, wall thickening in the RVFW and septum can also be found on the two cutting planes as shown in Fig. 8a. The RV P-V loops for the hybrid growth can be found in Fig. 8b. With additional concentric growth, the RV end-diastolic volume is less compared to the P&TR-Ecc case, which only allows eccentric growth in the RV, but with increased RV ESV. Thus, the RV pump function is not improved in this hybrid case with reduced SV. Both eccentric and hybrid growth cause RV P-V loops rightward shift with a much increased RV cavity volume compared to the baseline case. Figure 8c further compares the LV P-V loops, although LV SV stays the same for the two growth cases, the hybrid cases seems less hindering LV filling compared to the eccentric growth case, which could be explained by the less RV dilation as shown in Fig. 8b. Again, LV P-V loops shift leftward with a reduced SV compared to the baseline case. Our results seem to suggest that hybrid growth in RV may not lead to an improved pump function for this biventricle.

### Hybrid growth in the RV and the LV

**Fig. 9:**
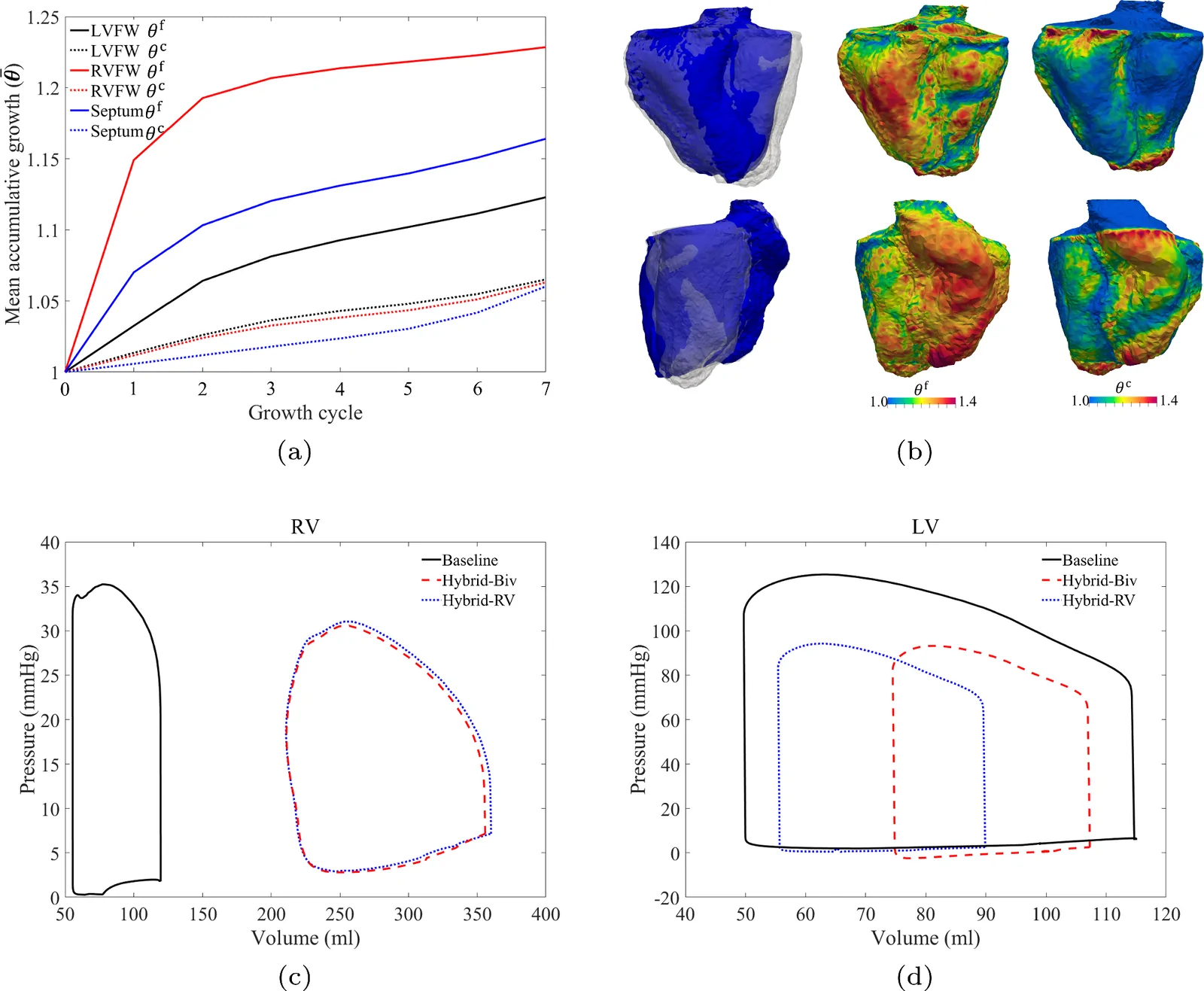
Comparison of hybrid G&R with and without LV G&R: (**a**) mean accumulative concentric ratio ($\theta ^\text {c}$) and eccentric growth ratio ($\theta ^\text {f}$) in the LVFW, RVFW and septum; **b** the final grown geometry with (grey) and without (blue) LV G&R, along with distributions of growth ratios with LV G&R after 7 G&R cycles; instantaneous pressure-volume loops for the RV (**c**) and the LV (**d**)

As can be found in previous sections, LV dynamics are significantly affected by PR and TR, further by RV growth. Here, we further explore how the heart adapts its structure and function if both the RV and LV experience hybrid growth. Figure 9a shows the mean accumulative growth amounts of $\theta ^\text {f}$ (eccentric growth) and $\theta ^\text {c}$ (concentric growth) in the RVFW, septum and LVFW over 7 growth cycles. Because growth is triggered by PR and TR, eccentric growth still dominates myocardial growth in the RV and LV, with some limited concentric growth. The largest growth is from the RVFW, followed by the septum, and the least in the LVFW, which could be explained by the fact that the primary response to TR and PR is from the RV. Moreover, because of the LV-RV interdependence and geometrical distortion caused by RV growth, the LV also experiences some growth, with $\theta ^\text {f} > 1$ on LV wall, although with much less wall thickening compared to the RV. Figure 9b further shows $\theta ^\text {f}$ and $\theta ^\text {c}$ at the 7$^\text {th}$ growth cycle. The P-V loops can be found in Fig. 9c for the RV and Fig. 9d for the LV. Compared to the hybrid-RV case, the RV pump function stays nearly the same and has the same level of significant RV dilation, while the LV P-V loop is less shifted leftward compared to the hybrid-RV case in relation to the baseline case. Although the LV EDV is larger compared to the hybrid-RV case, the increased LV ESV offsets such a change in diastole, thus no noticeable improvement in LV SV for the hybrid-Biv case. Compared to the baseline case, in fact, the LV pump function is still significantly reduced due to RV growth. Therefore, LV growth seems not to improve much on LV pump function for the hybrid-Biv case.

### Comparison between the grown RV with selected clinical studies

Table 3 summarises the RVEDV-to-LVEDV ratio, and RV eEF from the final grown configurations of all growth cases. The RVEDV-to-LVEDV ratios all increase compared to the values before the onset of growth. Śpiewak et al. (2012) found that a ratio between 1.70 and 1.82 indicates severe remodelling for patients with severe pulmonary and tricuspid regurgitation. In the final grown state, all ratios are greater than 2, and the highest ratio is from the P&TR-Ecc. eEF are all below the critical value identified by Zhang et al. (2026), and are also reduced from the onset of growth (see Table 2). Both RVEDV-to-LVEDV ratio and eEF suggest maladaptive remodelling and a higher risk of RV failure, particularly for the P&TR-Ecc case.

**Table 3:** Summary of RVEDV-to-LVEDV ratios and RV eEF values in the final grown states, with reported clinical ranges. Values in parentheses indicate the ratio prior to the onset of growth

cases	quantity	simulation	clinical ranges
PR-Ecc	RVEDV:LVEDV	3.12 (1.9)	1.74 Mauger et al. (2021)
	RV eEF(%)	15.52	10–54 estimated from Greutmann et al. (2022)
TR-Ecc	RVEDV:LVEDV	2.07 (1.5)	1.69−2.29 Zhang and Falco (2025)
	RV eEF(%)	24.00	$\le 25\%$ Zhang et al. (2026), 26.94% Zhang and Falco (2025)
P&TR-Ecc	RVEDV:LVEDV	4.37 (2.35)	1.0-− 4.5 Śpiewak et al. (2012)
	RV eEF(%)	10.26	$\le 25\%$ Zhang et al. (2026)
hybrid-RV	RVEDV:LVEDV	3.78 (2.35)	1.0-− 4.5 Śpiewak et al. (2012)
	RV eEF(%)	11.19	$\le 25\%$ Zhang et al. (2026)
hybrid-Biv	RVEDV:LVEDV	3.19 (2.35)	1.0-− 4.5 Śpiewak et al. (2012)
	RV eEF(%)	10.57	$\le 25\%$ Zhang et al. (2026)

**Fig. 10:**
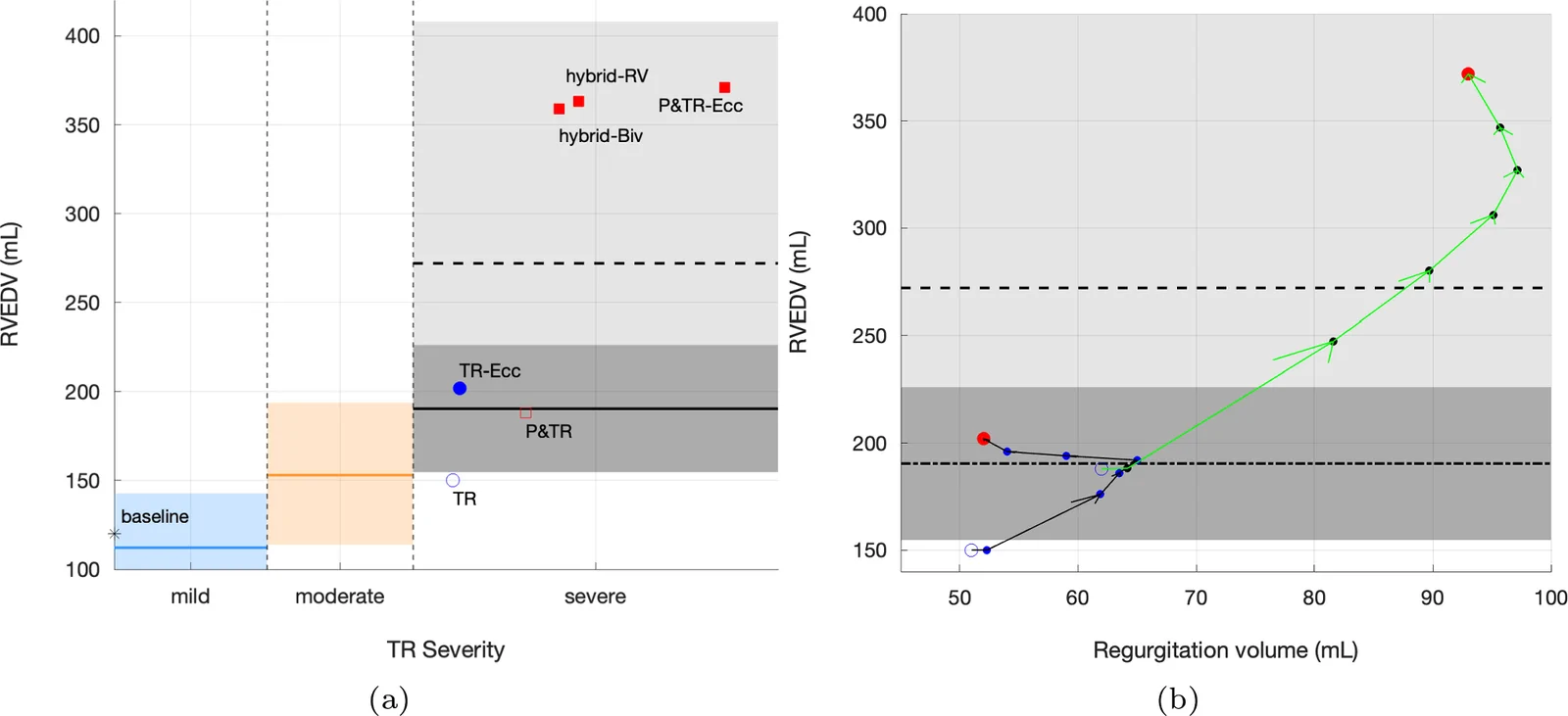
Comparison of simulated growth trajectories with clinical data from Caravita et al. (2025) and Greutmann et al. (2022). **(a)** RVEDV ranges for different TR severity groups (no/mild, moderate, and severe) reported by Caravita et al. (2025), with mean values indicated by solid lines, and the horizontal axis represents the extracted regurgitant volume for each group, mild: ≤23 ml, moderate: 23–45 ml, severe: ≥45 ml. Clinical data from Greutmann et al. (2022) are also included (light grey region), with mean values shown as dashed lines; (**b)** trajectories of RVEDV for the TR-Ecc (black) and P&TR-Ecc (green) cases, plotted within the severe group. Each arrow represents one growth cycle

Caravita et al. (2025) reported detailed RV haemodynamic measurements in patients with TR, categorised into three severity groups (no/mild, moderate, and severe). Figure 10 presents the corresponding ranges of RVEDV for these groups, with mean values indicated by solid lines. The TR severity classification is based on regurgitant volume as reported in Caravita et al. (2025). In addition, data from Greutmann et al. (2022) are included, overlapping with the severe group and shown as a light grey region with dashed mean values. As shown in Fig. 10a, both the TR and P&TR cases fall within the severe group at the acute state. Following growth, the TR-Ecc case remains closest to its acute configuration while still lying within the reported clinical ranges, indicating a relatively moderate progression compared to other cases. Figure 10b further illustrates the trajectories of the TR-Ecc and P&TR-Ecc cases starting from the acute state. Both cases exhibit progressive RV dilation, but with distinct growth trajectories, with the P&TR-Ecc case showing a more pronounced increase. Notably, the regurgitant volume decreases slightly after several growth steps, although it remains substantial. This reduction may be associated with diminished contractile function in the severely dilated RV, leading to reduced effective ejection and altered haemodynamics. These captured trajectories demonstrate that our model can reproduce clinically plausible pathways of RV dilation under sustained regurgitation.

## Discussion

This work introduces a subject-derived biventricular computational model that simultaneously incorporates pulmonary and tricuspid valve regurgitation and predicts long-term myocardial growth using an updated reference configuration framework. Unlike previous studies that focused on isolated valve dynamics or short-term haemodynamics, our approach captures the progressive structural adaptation of the RV and its impact on LV function. The novelty of this study lies in (i) integrating valve regurgitation mechanisms with mechanobiological growth laws in a unified finite element framework, (ii) quantifying maladaptive RV adaptation under combined pulmonary and tricuspid regurgitation, and (iii) demonstrating how RV adaptation alters LV geometry and pump performance.

The use of patient-specific biventricular models is becoming increasingly important in understanding cardiac function, particularly in the context of diseases affecting the RV, such as TOF (Arjoune et al. 2025), PR (Guan et al. 2023a), and TR (Lee et al. 2019), all of which contribute to adverse remodelling of the RV and may lead to heart failure. As a result, the RV has gained more focus in simulations of cardiac function in recent years. A patient-specific biventricular geometry is constructed with detailed fibre structures in the RV, LV, and RVOT following a rule-based approach. RV regurgitation is modelled by adding additional pathways from the pulmonary artery to the RV, and from the RV to the right atrium in the circulation model. This simple approach allows us to simulate the changes in pressure and volume between connected cavities. A similar boundary condition approach has also been used by other studies to model systemic circulation (Sack et al. 2018). The developed biventricular model captures key biomechanical features of RV function under PR and TR, including increased RV filling volume, elevated RV pressure, enhanced ventricular work, and leftward displacement of the interventricular septum. See a detailed comparison with literature values in Table 2. Both TR and PR are introduced abruptly to the baseline case, which may not always be the case in patients as chronic PR is typically a slow and progressive condition as seen in adult patients with repaired Tetralogy of Fallot, while acute PR could still happen due to a sudden structural disruption of the pulmonary valve leaflets. Acute TR is more common, for example due to chordae rupture.

Valve regurgitation typically leads to volume overload inside ventricles, causing eccentric growth by the addition of sarcomeres in series with progressive chamber volume (Grossman et al. 1975; Costabal et al. 2019). This adaptation is initially beneficial via the Frank-Starling mechanism by maintaining sufficient stroke volume. However, with persistent volume overload, the ventricular wall becomes thinner and an increase in wall stress according to the law of Laplace (Gsell et al. 2018). Elevated wall stress has been recognised as another mechanical stimulus to trigger concentric growth by adding sarcomeres in parallel, which tends to normalize the wall stress and increase wall thickness (Grossman et al. 1975). The concentric growth in our model emerges as a secondary response compared to the dominant eccentric growth, as shown in Fig. 8, resulting in almost no change in RV pump function despite some thickening of the RVFW. The reason for that could be because the regurgitation in RV is very severe, with a regurgitation fraction almost above 50%, RV remodels itself into a decompensated stage within 7 growth cycles with the chosen growth law, as the RV end-diastolic volume is 370 mL and the end-systolic volume 190 mL for the P&TR case, both volumes are far beyond the physiological range (Petersen et al. 2016): RV EDV 182±36 mL for male and 130±24 mL for female; RV ESV 85±22 mL for male and 55±15 mL for female, also beyond the ranges reported in Zhang and Falco (2025). The compensatory concentric growth will not reverse the adverse remodelling process that is already overwhelmed by eccentric growth, as suggested in Fig. 8. Clinical studies (Dietz et al. 2019) have demonstrated that significant TR usually leads to RV dilation and pump dysfunction with the worst outcomes, which suggests that maladaptive remodelling dominates RV remodelling under significant regurgitation, as also suggested by our models. Thus, immediate intervention is necessary to prevent or reverse such an adverse remodelling process.

The choice of growth trigger is very fundamental when modelling cardiac growth. End-diastolic fibre stretch has been used for driving eccentric growth, and the peak systolic stress has been used for driving concentric growth (Göktepe et al. 2010; Guan et al. 2023b). The stretch/stress based triggers have been widely adopted in the literature because they are primarily linked to the mechanical stimuli under volume and pressure overload (Costabal et al. 2019; Grossman et al. 1975). However, other biomechanical triggers could also be possible, such as shear strains, see Witzenburg and Holmes (2017) for a comparison of different growth laws with different biomechanical triggers. Recent studies have started to incorporate both mechanical and hormonal drivers for driving cardiac hypertrophy (Estrada et al. 2021; Xu et al. 2025). Such incorporation of bio-chemo-biomechanical triggers has the potential to capture a more realistic disease progression of cardiac growth in a truly multiscale way, thus enabling to identify critical pathways leading to adverse remodelling. Biomechanically, myocardial eccentric growth could be driven by fibre stretch during the passive filling phase, which has been successfully applied to model volume overload in the heart (Guan et al. 2023a). The mechanisms of PR and TR differ in how they lead to RV volume overload. PR occurs during the isovolumetric relaxation phase and early diastolic filling due to pulmonary valve dysfunction, which directly causes RV volume overload in diastole. In contrast, tricuspid valve dysfunction leads to blood being pumped back into the right atrium during the isovolumetric contraction and the ejection phases, resulting in initial volume overload of the right atrium. This subsequently leads to RV overload during the filling phase. Both conditions ultimately result in RV volume overload, resulting in higher stretch in diastole compared to physiological conditions. As a result, RV dilation caused by both PR and TR could be simulated using a growth law depending on fibre stretch as defined in Eq. (18). The growth triggers and their laws are phenomenological in this study; future work shall consider myocardial growth as a multi-factor pathological process.

Physiologically, eccentric and concentric growth stimuli may coexist. However, cardiac growth and remodelling is a complex and heterogeneous process, and the relative contribution of different mechanisms can vary depending on the underlying pathology, with no definitive evidence indicating that they are always activated simultaneously or to the same extent. In the case of pulmonary and tricuspid valve regurgitation, clinical studies consistently show that the dominant driver is right ventricular volume overload, which leads primarily to ventricular dilation and eccentric remodelling rather than wall thickening (Tadic et al. 2022; Śpiewak et al. 2020). Therefore, the eccentric-only simulations in this study are intended to isolate and characterise the primary adaptation mechanism relevant to these conditions. We did not consider a concentric-only scenario, as concentric hypertrophy is more typically associated with pressure-overload conditions (e.g. pulmonary hypertension) rather than regurgitation-driven volume overload. The hybrid growth case was included to address the potential coexistence of both mechanisms. Interestingly, our results demonstrate that even when both triggers are active, eccentric growth remains the dominant response in the RV, which is consistent with clinical observations of RV remodelling in PR and TR. Moreover, eccentric and concentric growths are treated independently without accounting for potential interactive contributions between the two growth processes.

The simulations without LV growth are intended to isolate the primary effects of RV remodelling under PR and TR. Some clinical studies found minimal changes in LV structure and function can exist despite significant RV remodelling (Slim et al. 2025). Consistent with clinical observations, our simulations with LV growth also show limited LV remodelling. While LV and RV are not independent pumps because they share the interventricular septum and are enclosed in the pericardium. Thus, changes in one chamber would inevitably impact the other chamber’s structure and function (Naeije and Badagliacca 2017). This ventricular interdependence implies that chronic RV volume overload, as induced by severe TR and PR, will hinder LV filling by lowering its effective compliance (i.e. leftward septal shift, restriction of LV filling space), and further depressing LV contraction without altering its intrinsic contractility (i.e. through reduced preload). As can be seen in Table 2, LV end-diastolic volumes are lower in the PR, TR and P&TR cases compared to the baseline case, with a similar trend for stroke volume, though ejection fractions are very close owing to decreased LV end-diastolic volume. The preserved ejection fraction suggests that LV contractility is not impaired in the acute regurgitant phase. Following RV growth over seven growth cycles, the LV end-systolic volume increases relative to the pre-growth state, as shown in Figs. 6d–f, suggesting a depressed LV pump function. Even allowing LV growth in the hybrid-Biv case, the LV pump function is still suppressed, see Fig. 9. Studies on rTOF patients have reported that severe PR and apical RV dilation are associated with both diastolic and systolic dysfunction in LV (Tretter and Redington 2018), leading to abnormal ventricular interaction and global hypokinesis (Demonceaux et al. 2025). Our results are consistent with those clinical observations and further support the notion that RV growth is inherently a biventricular process. This modelling paradigm provides a foundation for predictive simulations of RV disease progression. Once validated, particularly with respect to the rate parameters in the growth laws, the framework could be used to assess the outcomes of valve regurgitation correction. It could also help anticipate adverse remodelling and support the optimisation of intervention timing before irreversible RV failure develops.

The kinematic growth theory has been adopted to predict RV long-term response, while the adopted growth laws do not differentiate between different biological components within the heart wall, i.e. collagen fibres and myocytes. Although our models can capture ventricular dilation and predict biventricular dysfunction, such simplifications can not describe constituent-specific responses as done in constrained mixture models (Humphrey and Rajagopal 2002; Gebauer et al. 2023), especially the complicated interactions between collagen fibres and myocytes (Guan et al. 2025; Gebauer et al. 2023). Future work will extend this model with a constrained mixture model following (Guan et al. 2023b) by incorporating collagen turnover rates, myocyte hypertrophy to assess the risk of RV maladaptive remodelling under regurgitation. It would also enhance the model’s predictive capability for fibrosis, contractile dysfunction, etc. However, further measurements on patients’ heart conditions are required in order to achieve personalised modelling, such as the content of collagen fibres, myocyte architecture, and, in particular, the related longitudinal data.

In the present study, the remodelling tensor $\mathbb {P}_\text {r}$ is set to the identity, meaning that no additional rearrangement of tissue constituents is explicitly modelled. $\mathbb {P}_\text {r}$ could represent processes such as fibre reorganisation or collagen turnover that lead to incompatible intermediate configurations in addition to the growth tensor $\mathbb {G}$. Here, we focus on growth to capture the primary geometric adaptation of the myocardium under volume overload. Similar approaches have been applied widely in the literature (Göktepe et al. 2010). The choice of $\mathbb {P}_\text {r}$ is therefore a simplification to focus on growth and avoid introducing additional degrees of freedom that cannot be constrained by available data. Incorporating $\mathbb {P}_\text {r}$ would require further information on microstructural reorganisation, which is currently not well characterised under PR/TR and would introduce additional uncertainty into the model. We also note that the updated reference configuration framework itself does not require $\mathbb {P}_\text {r}=\boldsymbol{\textrm{I}}$, see Guan et al. (2023b).

After each growth step, the deformed fibre-sheet-normal system is re-orthogonalised to define the next reference configuration. This reorientation does not represent an explicit physiological remodelling law, nor a mathematical requirement of the adopted framework, but a simplification to maintain a physiologically consistent layered architecture. Experimentally, myocardial tissue has been shown to exhibit an approximately orthogonal fibre-sheet-normal structure across species and imaging modalities (e.g. LeGrice et al. 1995; Gilbert et al. 2012). However, there is currently limited data describing how this microstructure reorganises under pathological growth and remodelling. Therefore, rather than prescribing a specific biological reorientation mechanism, we assume that the orthogonal structure is preserved after each growth cycle. This assumption is also consistent with the adopted constitutive model, which is formulated based on this orthogonal system. More realistic models of fibre/sheet reorientation could be incorporated in the developed growth model when experimental data become available.

A simplified circulation model is adopted, which cannot describe the realistic blood flow like the one-dimensional model with rich arteries and veins. The parameters of the circulatory model are also kept constant during growth, with no systemic feedback mechanisms, such as baroreflex regulation, vascular adaptation. We acknowledge that these feedback processes can influence cardiac haemodynamics by modulating contractility, vascular resistance and cardiac output, and therefore would affect the quantitative values of pressure, volume, and wall stress. Specifically, in our simulations, LV end-systolic pressures remain within physiological ranges across all cases (Table 2), indicating that no strong baroreflex response would be expected. As a result, its influence on the growth and remodelling process in this study is likely to be limited, with growth predominantly governed by mechanical stimuli induced by regurgitation. Thus, we expect that the relative trends observed between different regurgitation scenarios would be less affected by the omission of systemic feedback mechanism compared to the absolute values of the predicted haemodynamics quantities.

The biventricular geometry was reconstructed at early-diastole, which was not necessarily unloaded when they experience the lowest ventricular pressure. In the literature, LV and RV geometries at different cardiac phases have been used to approximate the unloaded state, such as end-systole (Hadjicharalambous et al. 2021), early-diastole (Genet et al. 2014), mid-diastole or end-diastole (Feng et al. 2024). If the ventricular geometry is reconstructed at mid-diastole or end-diastole, an inverse approach will be applied to find out one possible unloaded state (Rausch et al. 2017). Additionally, Hadjicharalambous et al. (2021) suggested that an image-derived reference configuration, such as end-systole, is suitable for reliable comparative analyses in personalized cardiac modelling, provided that a consistent reference configuration is used across all models. In the baseline normal model, lowest LV and RV pressure occur at the beginning of diastolic filling, 2.02 mmHg in LV and 0.3 mmHg in RV. Due to the different operating pressure levels of the LV and RV, the exact timing of these minima is not strictly identical, nor expected. Future work should investigate how different reference configurations influence myocardial growth and remodelling process.

The PR and TR cases are not calibrated from real patient data but derived from a healthy biventricular model. Therefore, it would not be possible to validate the predicted growth patterns with real data. Instead, we have compared our simulated data extensively with clinical literature. In general, our simulated scenarios are consistent with clinical observations as shown in Tables 2 and 3, demonstrating the feasibility of predicting the trajectory of RV growth under regurgitation using a biomechanical growth model, see Fig. 10.

Other limitations include: (1) not considering mechanical constraints from the pericardium and surrounding tissues, which may play an important role and should be addressed in future work; (2) assuming identical growth behaviour for the septum and RVFW, which may not capture their potentially different adaptive responses; and (3) adopting simplified biomechanical growth triggers, based on the mean of the maximum fibre stretch during diastole and the maximum active tension during systole relative to the normal case. While these triggers are useful, they may not fully represent the complex and spatially heterogeneous responses of the myocardium.

## Conclusion

We have presented a biventricular biomechanics model that incorporates both pulmonary and tricuspid regurgitation, and we further combine a kinematic growth framework to study the longer-term myocardial adaptation due to chronic valve regurgitation in the RV. Simulation results suggest that regurgitation-driven volume overload could lead to dominant eccentric RV dilation with limited functional compensation from concentric thickening, and that RV growth could significantly compromise LV filling and systolic performance, underscoring the importance of ventricular interdependence in RV maladaptation. Furthermore, extensive comparisons with reported clinical ranges demonstrate that the simulated haemodynamic and functional responses are broadly consistent with clinical observations, and that the model captures clinically plausible trajectories of RV growth under sustained regurgitation. These findings suggest that biomechanical modelling of myocardial adaptation can provide mechanistic insights into RV adaptation under severe valve regurgitation and may support clinical decision-making once calibrated with individual patient data. Future work should focus on validating the myocardial growth laws using experimental and clinical data, and extending the framework to patient-specific scenarios for modelling of RV dysfunction.

## Data Availability

Supporting materials are available at https://github.com/HaoGao/Growth-Remodelling/tree/main/RV-Growth-Regurgitation.
